# Host traits and environmental factors shape infection heterogeneity in wild rat–protozoa networks

**DOI:** 10.1093/ismeco/ycag026

**Published:** 2026-02-10

**Authors:** Matan Markfeld, Itamar Talpaz, Barry Biton, Toky Maheriniaina Randriamoria, Voahangy Soarimalala, Steven Michael Goodman, Charles L Nunn, Georgia Titcomb, Shai Pilosof

**Affiliations:** Department of Life Sciences, Ben-Gurion University of the Negev, Be’er-Sheva, 8410501, Israel; Department of Life Sciences, Ben-Gurion University of the Negev, Be’er-Sheva, 8410501, Israel; Department of Life Sciences, Ben-Gurion University of the Negev, Be’er-Sheva, 8410501, Israel; Association Vahatra, Antananarivo, B.P. 3972, Madagascar; Association Vahatra, Antananarivo, B.P. 3972, Madagascar; Institut des Sciences et Techniques de l’Environnement, Université de Fianarantsoa, Fianarantsoa, 301, Madagascar; Association Vahatra, Antananarivo, B.P. 3972, Madagascar; Field Museum of Natural History, Chicago, IL, 60605, United States; Department of Evolutionary Anthropology, Duke University, Durham, NC, 27708, United States; Duke Global Health Institute, Durham, NC, 27708, United States; Department of Fish, Wildlife, and Conservation Biology, Colorado State University, Fort Collins, CO, 80521, United States; Department of Life Sciences, Ben-Gurion University of the Negev, Be’er-Sheva, 8410501, Israel; The Goldman Sonnenfeldt School of Sustainability and Climate Change, Ben-Gurion University of the Negev, Be’er Sheva, 8410501, Israel

**Keywords:** Host–microbe network, Infection heterogeneity, Land-use change, Machine Learning, Stochastic block modeling

## Abstract

The occurrence of microbes in animal hosts is highly heterogeneous, shaped by interactions among host traits, environmental context, and microbial diversity. Understanding this heterogeneity is particularly critical for endoparasite infections, where some hosts harbor diverse, high-burden assemblages that elevate disease spread and spillover risk. Yet the mechanisms underlying such heterogeneity remain poorly understood in wild systems, especially at the individual-host level. We addressed this challenge by studying protozoan infections in introduced black rats (*Rattus rattus*) across environmental gradients in Madagascar. Using network-based stochastic block modeling, we identified three infection profiles capturing meaningful variation in protozoan richness and composition, providing a structured framework for understanding heterogeneity. To uncover the predictors of these profiles, we trained machine-learning models incorporating host traits with environmental variables. Our models consistently outperformed no-skill baselines, with host traits contributing $\sim$40% more to predictions than environmental factors. Body mass and gut microbiome composition emerged as the strongest host predictors, while rat and other non-native species densities were the most influential environmental predictors. These results show that infection heterogeneity arises from the interplay of intrinsic host traits and extrinsic environmental conditions. Our approach illustrates how combining network analysis with predictive modeling can (i) uncover latent heterogeneity in host–microbe associations, (ii) identify the relative contribution of the factors driving this heterogeneity, and (iii) predict host infection profiles. Our framework advances microbial ecology by linking host traits, microbial communities, and environmental context, while also informing disease ecology at human–animal interfaces where zoonotic pathogens circulate.

## Graphical Abstract

**Graphical Abstract:**
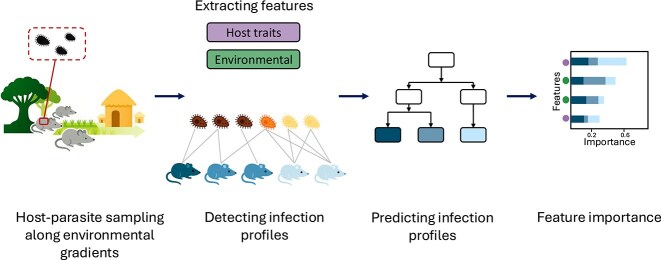

## Introduction

Heterogeneity in microbe occurrence across hosts is a typical characteristic of microbial systems [1, 2]. Specifically, in microbial host–parasite systems (e.g. protozoans, viruses, and bacterial pathogens), parasite distributions are typically skewed: most hosts harbor few parasites, while a small subset carries heavy burdens [2–4]. This variation, driven by differences in host physiology and environmental context, has major consequences for transmission dynamics, infection persistence, and spillover risk [5–9]. Further complexity arises from co-infections involving multiple parasite species or strains, each with distinct natural history traits and transmission strategies [10–12]. Understanding these interacting sources of heterogeneity is essential for predicting and mitigating parasite spread and impact [13]. However, the combined influence of host traits, co-infections, and environmental factors on patterns of parasite infection heterogeneity remains poorly understood, particularly at the individual host level.

Bipartite networks, where parasites are linked to the hosts they infect, offer a powerful framework for studying heterogeneity in host–parasite interactions [14]. However, studies to date have largely focused on species-level networks to explore ecological and evolutionary processes underlying heterogeneity [15–18], with only a limited use of individual-level networks [19]. Individual-level networks can be used to identify groups of individual hosts with similar parasite associations and, conversely, groups of parasites infecting hosts with similar characteristics. These emergent group structures, which we term *infection profiles*, can provide insights into the ecological and epidemiological roles of hosts and parasites. Examples include distinguishing generalist parasites from rare or highly specialized ones [20], or identifying host groups with distinct parasite assemblages.

Detecting infection profiles involves clustering nodes with similar interaction patterns, which can be done using stochastic block models (SBM) [21]. Unlike clustering or pairwise approaches that emphasize dense within-group links or node-level similarity, SBMs identify latent groups based on equivalence in connectivity patterns, capturing similarities in the probabilities of connections both within and between groups across the network [21, 22]. SBM is an unsupervised clustering approach (the number of groups and their composition is inferred rather than predetermined), explicitly designed for network data and applied simultaneously to both hosts and parasites. Despite their potential [23], SBMs are rarely used in ecological analysis. However, previous studies confirmed their usefulness [24–27]. For example, in the human gut microbiome, SBMs identified generalist and specialist microbes [25]. To date, however, SBMs have not, to our knowledge, been applied to host–parasite networks.

Here, we identify infection profiles in individual hosts of the introduced black rat (*Rattus rattus*) and their protozoan parasites in northeastern Madagascar. Protozoa commonly inhabit the mammalian gut, yet few studies have explored their diversity in wild mammals or the factors influencing their community structure [28]. Detecting infection profiles is particularly important in rural, low-income regions like Madagascar, where introduced rats, living both in the wild and near human settlements, serve as reservoirs for zoonotic pathogens, increasing the risk of spillover to humans [29, 30]. For example, in Madagascar *R. rattus* was previously found to have an infection rate of $\sim$20%–50% for protozoa of the zoonotic genera *Trypanosoma, Cryptosporidium*, and *Giardia*, which have also been detected in humans on the island [30, 31].

While infection profiles can reveal structural heterogeneity in host–parasite interactions, they do not explain how it emerges. To gain mechanistic insight into these interactions, our second goal was to identify the intrinsic and extrinsic factors shaping infection profiles by focusing on the two main phases of infection: exposure (the likelihood of encountering a parasite) and susceptibility (the likelihood of infection postexposure) [32–34]. Intrinsic host traits such as sex, age, body mass, immune function, and co-infections can influence both stages by affecting host behavior, immunity, and survival [35–37]. The host microbiome is also a key modulator, shaping susceptibility via its effects on immunity, metabolism, and overall health [38, 39]. Extrinsic factors, including land-use change, habitat fragmentation, and environmental reservoirs, alter host movement, contact rates, and exposure risk [40–43]. For example, gut protozoa, transmitted via fecal–oral routes, are particularly sensitive to environmental contamination, which varies in different ecological contexts [44, 45]. These extrinsic pressures often interact with intrinsic traits—for instance, through effects on diet, body condition, or stress. However, how such interactions drive infection heterogeneity remains poorly understood, especially in wild or semi-wild mammal populations.

**Figure 1.**
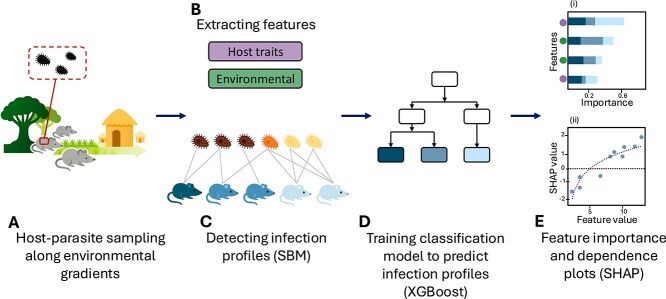
Conceptual scheme of methods. (A) *R. rattus* individuals and their protozoan parasites were sampled along a land-use change gradient to investigate infection heterogeneity. (B) Host traits (e.g. mass, sex, nematode co-infection status, and microbiome composition) and environmental features (e.g. habitat attributes and population density) were measured for each host. (C) We used Stochastic Block Modeling (SBM) to identify host and parasite infection profiles based on interaction patterns in the host-parasite network. Colors indicate SBM-assigned group membership of protozoa OTUs and rat hosts. (D) We trained an XGBoost model on the host and environmental features to quantify their relative importance in predicting infection profiles. (E) (i) We used SHAP values to estimate the mean absolute contribution of each host-trait (purple circles) and environmental (green circles) feature by measuring changes in model predictions upon feature removal [70]. (ii) The relationship between features (e.g. host mass) and SHAP values for each infection profile was assessed. Positive SHAP values indicate a feature that supports classification into an infection profile, while negative values indicate the opposite .

We combined SBM and machine learning with detailed field sampling of ecological and biological data to detect infection profiles and assess the key drivers of infection heterogeneity. Our dataset is unusually rich and detailed, containing diverse host traits (e.g. body mass, sex, nematode co-infection, and gut microbiome composition) and environmental factors (e.g. habitat characteristics, disturbance, and community composition). This allowed us to test multiple host and environmental drivers simultaneously, an approach rarely possible in most systems. By linking these factors to SBM-identified infection profiles, we disentangle the relative contributions of host traits and environmental conditions to emerging heterogeneity in parasite infection patterns (Fig. 1).

## Materials and methods

### Study site and sampling

Our study focused on the black rat (*R. rattus*), a species introduced to Madagascar probably during the 10^th^-century and the most abundant small mammal in non-forested, rural areas on the island [46–48]. As a large generalist omnivore with a highly adaptable diet [49], *R. rattus* is a primary agricultural pest, a major disruptor of native ecological communities, and a potential vector for zoonotic diseases in the area [29, 48].

Rats were collected in the vicinity of three villages (Mandena, Sarahandrano, and Andatsakala) in the SAVA Region of northeastern Madagascar, in the area surrounding Marojejy National Park (SI note 1). The park consists of natural moist evergreen forests spanning a wide elevation range, from lowland areas to mountain peaks exceeding 2000 m. At each of the three villages, small mammals were sampled across seven habitat types (sites) representing a degradation gradient: (i) semi-intact natural forest inside the national park, (ii) secondary forest, (iii) *savoka* (brushy regrowth), (iv) agroforest (vanilla plantation), (v) mixed agriculture (sugarcane/coffee plantation), (vi) flooded rice fields, and (vii) village. Sites in each village setting were located $\sim$500 m apart. At each site, a grid of 121 live traps (arranged in an $11\times 11$ configuration with 10 m spacing) was deployed, supplemented by two pitfall trap lines outside the grid, each containing 11 buckets. During the study period, each site was sampled for six consecutive nights during three different seasons. Sampling was conducted at Mandena between October 2019 and September 2020, at Sarahandrano between November 2020 and September 2021, and at Andatsakala between October 2021 and August 2022.

### Protozoa DNA extraction and lab work

We used DNA metabarcoding to detect a range of protozoa in rats, identify nematode infections, and characterize the rat microbiome. Approximately 1 g of feces was preserved in either nucleic acid preservation (NAP) buffer [50] or Zymo DNA/RNA Shield (Zymo Research, Irvine, California). Two different storage solutions were used due to complications with lab supplies during the COVID-19 pandemic. Mean sequencing read abundance did not differ between the two sample types. DNA was extracted from fecal samples using Zymo MiniPrep Fecal kits (Zymo Research, Irvine, California) according to manufacturer directions.

We performed PCR with the G4 primer set [51, 52] to amplify 18S ribosomal DNA from a wide range of eukaryotes in the rat fecal DNA extracts. Forward and reverse primers contained 8-nucleotide barcodes with a Hamming distance of at least 4. PCR reactions were carried out in 15$\mu$L volumes consisting of: 3 $\mu$L of each forward and reverse primer (2 $\mu$M stock concentration); 7 $\mu$L from a Mastermix comprised of 0.7 $\mu$L of Amplitaq Gold polymerase, 150 $\mu$L MgCl2, 150 $\mu$L Amplitaq Gold buffer, 12 $\mu$L BSA, 6 $\mu$L DMSO, and 344 $\mu$L water; and up to 2 $\mu$L template DNA (1–100 ng total). Cycling conditions were: 10-min hot-start activation, 35$\times$ cycles of 15 s at 95$^{\circ }$C, 30 s at 57$^{\circ }$C, 40 s at 72$^{\circ }$C, and a final 5-min extension at 72$^{\circ }$C. DNA concentrations were then measured, pooled, normalized, and purified using MinElute columns prior to multiplexing with additional libraries. The final library for each village was sequenced three times on an Illumina MiSeq (v3 2 $\times$ 300 bp, 25 M reads) at the UC Davis Genome Center. Sequences were demultiplexed using cutadapt (v.3.4) with zero error tolerance [53]. We used the *dada2* bioinformatics pipeline [54] to filter and trim amplicons (minimum length = 100, 15% PhiX removed), remove errors, dereplicate, infer amplicon sequence variants (ASVs) using the pseudo-pooling method, merge pairs, remove chimeras, and combine the three ASV read tables from the different villages into one table.

### Bioinformatics and protozoa OTU processing

We calculated the relative read abundance of each ASV and excluded reads that accounted for <1% of a sample’s relative read abundance to avoid potential sequencing errors or tag jumps. We excluded any sample with fewer than 500 total reads due to potential amplification or sequencing failure ($n=38$). Due to less certainty in protozoal identifications, we then used a consensus approach to assign taxonomy to ASVs: we used both the “assignTaxonomy” function in *dada2* (minimum bootstraps = 50) and the “IdTaxa” function in the *DECIPHER* package in R [55] to generate two identifications for each ASV using the SILVA non-redundant database clustered at 99% similarity (v.132). For any ASV with a mismatching identification, we queried the sequence in the NCBI GenBank database. The final “consensus” ID was determined by selecting the identification with the highest sequence similarity to a reference sequence in GenBank, thus establishing the most reliable taxonomic label.

Next, we clustered phylogenetically similar ASVs into OTUs at 97% similarity using the “Clusterized” function from the DECIPHER package. For each OTU, the taxonomic assignment at the highest achievable resolution (genus or species level) was determined by the taxonomy associated with its most common ASV. Because the G4 primer set amplifies both parasitic and non-parasitic eukaryotic DNA, we then manually filtered all OTUs to those that are known or suspected parasites of mammals, using reference literature and taxonomic databases. Consequently, even when the taxonomic assignment was limited to the genus level, our protozoan community was analyzed at the OTU resolution, as these units capture the fine-scale genetic variations that may directly influence infection. Host–parasite network studies exclude uninfected individuals because the lack of a link could be a false negative. Our primer set amplifies both parasitic and non-parasitic eukaryotic DNA, allowing us to designate rats with only non-parasitic protozoa as “uninfected.” We still acknowledge that, as in any study, very low-abundance parasite infections might escape detection. In total, our dataset includes 841 individual rat hosts and 41 protozoan OTUs spanning 10 genera and 4 phyla (Fig. S2).

### Network construction and detection of infection profiles

We constructed a bipartite network representing individual rat hosts and protozoa OTUs, where an edge is present ($1$) if an OTU infects a host and absent ($0$) otherwise (Fig. 1C). Then, we identified infection profiles using SBM [56]. In the bipartite version of the SBM, hosts are assigned to $Q^{(1)}$ groups and protozoa parasites to $Q^{(2)}$ groups. The interactions between these groups are governed by a block connectivity matrix $\Theta$, which encodes the probability of infection between each pair of host and parasite blocks [56]. Consequently, the probability that a link exists between a host $i$ and a parasite $j$, which belong to groups $c_{x}$ and $c_{y}$, respectively, is $P_{ij} = \Theta{c_{x} c_{y}}$. This structure implies that nodes within the same block are “stochastically equivalent” and thus have similar probabilities of being infected by parasites from a given parasite block. Because SBM is based on links, we predefined uninfected hosts as a separate group and conducted the SBM analysis exclusively on the infected hosts.

We implemented SBM using a commonly used Variational Expectation–Maximization algorithm that iteratively estimates latent memberships and model parameters [56]. The algorithm partitions the network into groups and calculates the likelihood of such clustering, considering the membership of nodes in groups. The algorithm consists of two main steps: (i) updating the posterior probabilities of host and parasite assignments to latent blocks and (ii) optimizing model parameters—including the block connectivity probabilities $\Theta$—to maximize the likelihood of the observed data. To determine the optimal number of host and parasite blocks, we used the integrated completed likelihood criterion, which balances model fit and complexity by penalizing model size. This approach ensures robust and interpretable clustering [56].

### Feature collection and processing

To investigate the determinants of parasite infection patterns, we measured a set of host traits and environmental variables for each host (Fig. 1B, Table S1, and see SI note 2 for detailed explanations). These variables might influence infection patterns by affecting both host exposure and susceptibility to infection. We assessed nine host variables: (i) body mass, (ii) body condition, calculated by Body Condition Index, (iii) sex, (iv) age, categorized as subadult and adult, (v) nematode co-infection, measured as presence/absence of nematodes, and (vi–ix) gut microbiome composition, measured as the relative abundance of four microbial families that were significantly correlated with the first two principal coordinates in a PCoA (Fig. S9). Host mass, body condition, sex, and age can influence behavior (e.g. home range and social interactions) as well as physiological traits [57–59]. Co-infection with macroparasites can further influence infection with microparasites. Specifically, nematodes are known to modulate the host immune system, potentially altering susceptibility to co-infection by other parasites [37, 60]. The gut microbiome plays a critical role in host metabolism and immune function, serving as an indicator of overall health [39, 61]. Variation in microbial composition and relative abundance has previously been linked to several diseases and may reflect either an increased vulnerability of the host or a response to the disease itself [62, 63].

We measured six environmental variables: (i and ii) habitat structure (obtained via the first two PCs of a vegetation PCA that distinguishes between tree-dominated and herbaceous-dominated sites; Fig. S6), (iii) distance to the village center (a proxy for habitat disturbance), and (iv–vi) small mammal community composition (population densities of rats, native species, and other non-native species, including shrews and house mice). Population densities were calculated as the average number of individuals captured per sampling trap for every site and season. Vegetation type and proximity to the village are covariates that can influence the composition and survival of parasites in the environment, thus altering exposure risk to rats [43, 64]. Additionally, small mammal population density can impact infection patterns, as higher contact rates in denser populations may facilitate parasite transmission within and between host species [40, 42]. Thus, differences in species densities across sites may lead to distinct parasite transmission patterns among rat populations. The correlation between pairs of features ranged between −0.71 and 0.67 with an average absolute value of 0.13 (Fig. S3).

### Training a classification model to predict infection profiles

Our aim was to predict and explain membership in SBM-derived infection profiles based on multiple predictors. This task requires a methodology suited to high-dimensional, heterogeneous predictors and complex nonlinear interactions. Machine learning methods effectively capture such non-linear relationships, which traditional linear models cannot represent [65, 66]. We therefore adopted a flexible, predictive classification framework called XGBoost (eXtreme Gradient Boosting), which is a distributed decision tree machine learning algorithm based on gradient boosting that efficiently handles structured data and captures complex patterns through ensemble learning [67]. XGBoost is particularly well suited to our objective, in contrast to parametric multinomial mixed-effects models that are primarily designed for causal inference under linear modeling assumptions and for individual parasites. We trained an XGBoost multi-classification model to predict host infection profile membership (SBM-identified groups) using both host traits and environmental variables as suites of features (Fig. 1D).

To ensure that all data points were tested at least once while mitigating overfitting and improving the robustness of the analysis, we implemented a stratified nested cross-validation approach, maintaining class distributions across splits. The outer loop used a three-fold cross-validation, where the dataset was split into three equal parts, and each subset was used once as a test set while the remaining two served as the training set. Within the inner loop, we conducted a five-fold cross-validation for hyperparameter tuning. Hence, within each training set, the data were further divided into five subsets, with four used for training and one for validation. This process was repeated for each fold, optimizing hyperparameters across different partitions of the data. To address data imbalance (uneven sizes of the host infection profiles) and prevent the model from being biased toward the majority profile, we applied host profile weights inversely proportional to profile frequencies, assigning higher weights to minority profile samples during model training.

For hyperparameter tuning, we performed a grid search over key parameters, including maximum tree depth, learning rate (eta), column and row subsampling rates, L1/L2 regularization parameters (alpha and lambda), and minimum child weight (Table S2). The selected parameter search space aimed to minimize model complexity, thereby reducing the probability of overfitting. For each configuration, we trained the model using 300 boosting rounds, with early stopping based on validation loss to prevent overfitting. In each outer loop iteration, the best-performing hyperparameters (selected based on multi-class log-loss) were used to train a model, which was then evaluated on the outer test dataset. This process produced three models, corresponding to the three outer loops, each predicting a different fold of the dataset. The output of each model was a probability distribution over possible infection profiles, constrained to sum to one. The predicted profile was determined as the one with the highest probability.

To evaluate performance, we used a range of metrics designed to capture different aspects of predictive ability [68, 69] (SI note 3). Since we had three infection profiles (see *Results*), we used metrics for multi-class classification. The evaluation was based on a $3\times 3$ confusion matrix, which recorded the number of true positives and false positives for each infection profile. We used common evaluation metrics such as accuracy, weighted precision, weighted recall, weighted F1-score, weighted balanced accuracy, and Matthews correlation coefficient (MCC). To benchmark model performance against chance, we analytically derived expected metric values for a random classifier using proportional guessing based on profile prevalence. In addition, for each infection profile, we assessed one-vs-all performance across decision thresholds using Area Under the Receiver Operating Characteristic Curve (ROC-AUC) and Area Under the Precision-Recall Curve (PR-AUC). This allowed us to evaluate the model’s ability to distinguish among profiles and capture precision–recall tradeoffs, especially in the context of class imbalance. See SI note 3 for detailed explanations.

### Feature importance analysis

To interpret the contribution of each host or environmental variable to the classification task, we employed both XGBoost’s gain metric and SHapley Additive exPlanations (SHAP) values (Fig. 1E). SHAP explains machine learning model predictions by quantifying the contribution of each input feature. Specifically, SHAP values measure how much each feature increases or decreases the model’s prediction compared with the average prediction [70]. To determine these values, SHAP evaluates the model’s output across different subsets of features and calculates the difference in predictions when a feature is included versus when it is omitted, then averaging these differences across all possible feature combinations. This ensures that the contribution of each feature is measured while accounting for interactions with other features. The magnitude of a SHAP value represents the strength of a feature’s influence on the prediction, with larger absolute values indicating a greater impact.

We assessed global feature importance by averaging absolute SHAP values across all hosts in the test subset of the three-fold models (totaling 841 samples), and then summing these mean values across all infection profiles. Additionally, we aggregated feature importance by category (host traits versus environmental) by summing SHAP values within each category, enabling a comparative assessment of their relative influence on classification. We then used the SHAP values themselves to create dependence plots capturing how each feature’s effect varied across its observed range (e.g. the range of host masses), illustrating potential relationships with the profiles. Positive SHAP values indicate that a feature increases the prediction compared with the baseline model output, “pushing” the model towards a specific infection profile. In contrast, negative SHAP values decrease the prediction compared with the baseline, moving the model away from predicting that infection profile.

### Code and software

All analyses were conducted in R (v4.2.1) [71]. We used the R package *blockmodels* (v1.1.5) [56] for the SBM analysis, the package *XGBoost* [67] for model training, with the package *caret* [72] for cross-validation and performance evaluation. ROC and PR curves were created using the *pROC* [73] and *PRROC* [74] packages. SHAP values were computed with the package *shapviz* [75].

## Results

### Stochastic block modeling reveals structured host and protozoan profiles

The rat–protozoa bipartite network included 841 host nodes—271 uninfected (singleton nodes with no links) and 570 infected—and 41 protozoan OTU nodes. The network contained 1557 links, with a connectance of 0.045 (i.e. 4.5% of all possible links were realized).

**Figure 2.**
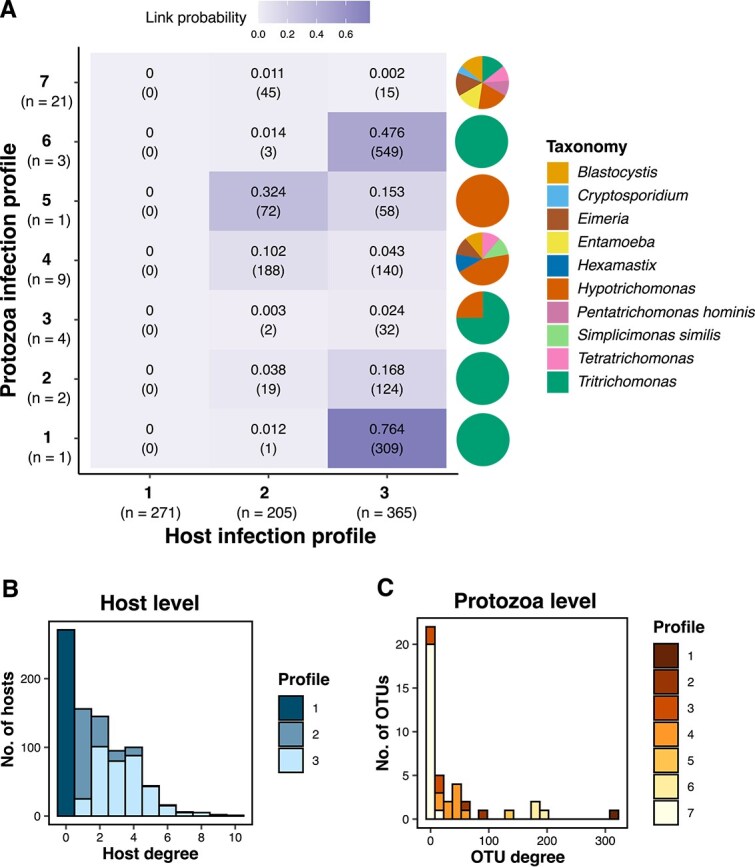
Infection profiles of *R. rattus* individual hosts and protozoan OTUs. (A) The block connectivity matrix $\Theta$ resulting from the SBM analysis of the rat-protozoa network. Rows and columns represent protozoa and host groups, respectively ($n$ indicates the number of OTUs or hosts in each group). Cell color reflects the SBM-predicted link probability between a host and an OTU that belong to certain groups. The link probability is denoted in each cell and the total number of observed links between groups is shown in parentheses. Pie charts depict the proportion of OTUs from each protozoan taxa (at the species/genus level) within each protozoan infection profile. (B) Host degree distribution (protozoan OTU richness with which a host is infected). (C) Protozoa OTU degree distribution (number of host individuals an OTU infects). Colors indicate protozoa and host infection profiles (SBM groups). The bars in (B) and (C) are stacked.

We identified two host groups based on the SBM analysis. Together with the uninfected group, this resulted in three *host infection profiles*. We also identified seven *protozoan infection profiles* (Fig. 2A, Fig. S1). The host infection profiles varied in size, with 271 individuals classified into the first profile, 205 into the second, and 365 into the third. Protozoan infection profile size ranged from a single OTU to 21 OTUs. We also found variation in host node degree. Specifically, rats from the first profile were infected by 0 protozoa OTUs (the uninfected profile), rats from the second profile were infected by 1–7 (mean = 1.61) OTUs, and rats from the third profile were infected by 1–10 (mean = 3.36) OTUs (Fig. 2B). The protozoan infection profiles also varied in degree, as OTUs from profiles 1, 5, and 6 (all are of the genera *Tritrichomonas* or *Hypotrichomonas*) were more prevalent, infecting 15%–36% of hosts, whereas OTUs from profiles 2–4 and 7 were rarer, infecting only 0.3%–8% of hosts (Fig. 2C, Fig. S2).

The host infection profiles differed not only in node degree but also in connectivity patterns, as captured by the block connectivity matrix $\Theta$ (Fig. 2A). Hosts in profile 1 were uninfected and showed no associations with any protozoan OTUs. In contrast, host infection profile 2 was characterized by infection with the prevalent *Hypotrichomonas* OTU. This was indicated by strong connectivity to protozoan profile 5, along with sporadic infections with diverse, low-prevalence OTUs (protozoan profiles 4 and 7). Host infection profile 3 exhibited higher overall infection levels, with particularly strong associations to the highly prevalent *Tritrichomonas* OTUs (protozoan profiles 1, 2, and 6).

Heterogeneous connectivity patterns were also found for the protozoa profiles, as some maintained a consistently low infection rate (e.g. profile 7), while others exhibited strong preferences to particular host infection profiles (e.g. parasite profiles 1, 5, and 6). Several protozoan profiles consisted of specific taxa (profiles 1–3, 5, 6, and 8 were mostly *Tritrichomonas* and *Hypotrichomonas*), while profiles 4 and 7 contained diverse taxa.

Overall, these emerging connectivity patterns demonstrate that the SBM effectively captured infection profiles, revealing latent heterogeneity in host–parasite interactions that reflects differences in parasite prevalence, host infection burden, and interaction specificity.

### Host traits outweigh environmental features in predicting host infection profiles

Our XGBoost model consistently outperformed a no-skill classifier in predicting host profiles (weighted precision = 0.53, weighted recall = 0.54, weighted F1-score = 0.53, weighted balanced accuracy = 0.64, and MCC = 0.28; SI notes 3 and 4). To evaluate the model’s ability to distinguish among profiles, we further assessed one-vs-all performance across decision thresholds. The ROC-AUC exceeded the random expectation of 0.5 for all profiles (ROC-AUC = 0.726, 0.606, 0.738, for profiles 1, 2, and 3, respectively). Similarly, the PR-AUC was above the no-skill baselines for the three host profiles (PR-AUC = 0.548 [no-skill of 0.322] for profile 1; PR-AUC = 0.331 [0.244] for profile 2; and PR-AUC = 0.656 [0.434] for profile 3). Therefore, we can predict infection profiles based on the features we selected.

**Figure 3.**
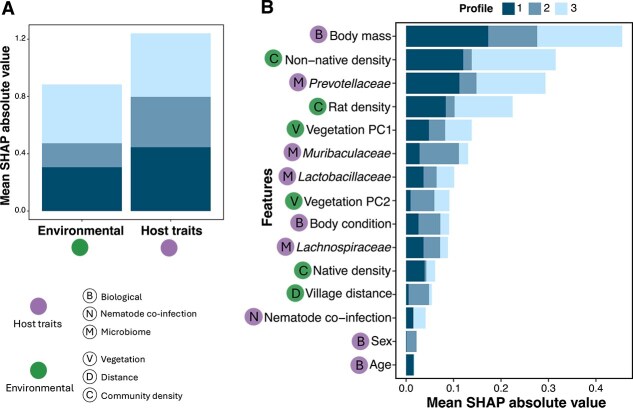
Importance of host traits and environmental variables in predicting host infection profiles. (A) Mean absolute SHAP values for host and environmental features across all host infection profiles, highlighting their relative contributions. (B) Feature importance, measured as the mean absolute SHAP values across hosts for all infection profiles. Features are ranked from most important (top) to least important (bottom). In both panels, bar colors depict host infection profiles. Circles indicate host (purple) and environmental (green) features, with the letter inside each circle depicting the corresponding subcategory of the feature per the legend at the bottom-left (Table S1) .

However, not all features contribute equally to prediction. To identify the features driving model predictions of host infection profiles, we used SHAP analysis, which quantifies each feature’s contribution to classification outcomes [70]. To assess the relative importance of host traits versus environmental factors, we summed absolute SHAP values within each category. While both categories of features influenced model predictions, host traits’ features contributed 40% more than environmental features (1.24 compared with 0.88 mean absolute SHAP) (Fig. 3A). This trend was consistent across all host infection profiles, with host features consistently showing higher absolute SHAP values. However, for host profile 3, the environmental features contributed almost as much as the host traits. The pattern held even when we considered only the top six host features to match the number of environmental features, with host features still exhibiting higher mean absolute SHAP values (1.16 versus 0.88).

The most influential host features included host body mass and the relative abundance of the gut microbial families *Prevotellaceae* and *Muribaculaceae* (Fig. 3B). Body mass is known to affect infection risk, and it is often linked to body condition, age, and sex [57, 59, 76–78]. However, these later features ranked low in their importance. Salient environmental features included rat and other non-native species densities at a site, together with the site’s vegetation structure (PC1) (Fig. 3B). Notably, the relative importance of all features varied among host infection profiles, with some playing a crucial role in predicting certain profiles while being less relevant for others. For example, rat density was an important feature in predicting host infection profiles 1 and 3, whereas its contribution to the prediction of profile 2 was lower (Fig. 3B).

### Host traits and ecological gradients structure host infection profiles

**Figure 4.**
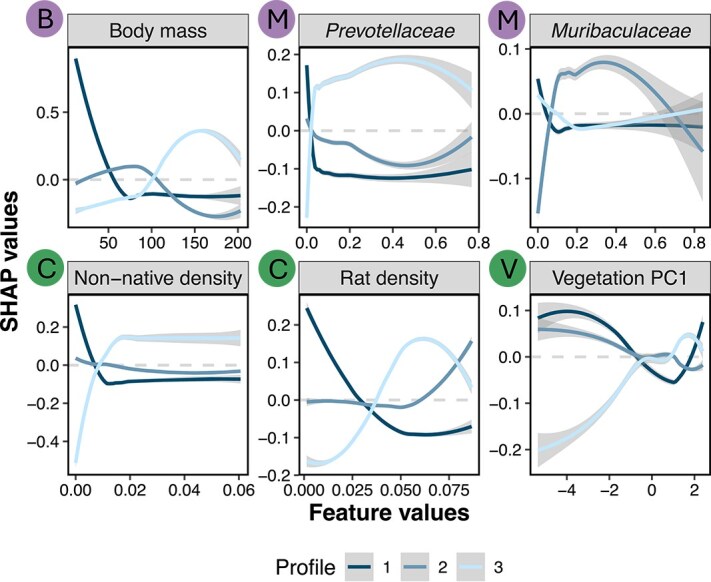
Key host traits and environmental factors predict infection profiles with distinct trends in SHAP values. SHAP dependency plots show individual hosts’ SHAP values (y-axis) as a function of feature values (x-axis). For clarity, we visualized only the LOESS-derived trend line and its associated confidence interval for the 841 host samples, rather than displaying all individual host data points. Line colors indicate host infection profiles. The dashed gray line marks a SHAP value of zero (i.e. no predictive value). LOESS line above and below 0 indicates that the classification of a profile is more or less likely, respectively. The top and bottom rows present the three most important host and environmental features, respectively ([B], Biological; [M], Microbiome; [C], Community density; [V], Vegetation).

To further investigate how the most important features influence the prediction of specific host infection profiles, we used SHAP dependency plots, which visualize the relationship between feature values and SHAP values. For most features, we observed clear trends between feature values and model predictions of infection profiles (Fig. 4, Fig. S4). For example, rats with a body mass <100 g were consistently classified into infection profile 1, which is associated with no infection, whereas larger rats were more often assigned to host profile 3, characterized by higher infection richness. Particularly interesting features were the relative abundance of the bacterial families *Prevotellaceae* and *Muribaculaceae*. A high relative abundance of *Prevotellaceae* in a host was influential in distinguishing host profile 3 (high infection). In contrast, a high relative abundance of *Muribaculaceae* was associated with host profile 2 (low infection) (Fig. 4). These bacterial families have been shown to play key roles in digestion, immune regulation, and gut health [79–81].

Among the environmental features, higher non-native species and rat densities were associated with a marked shift in predicted host infection profiles from 1 (no infection) to 3 (higher infection richness). This pattern was also evident along the vegetation gradient: negative values of vegetation PC1—indicative of less disturbed sites—were more associated with host profiles 1 and 2, and less so with profile 3. However, some features showed weak or no clear trends with specific infection profiles (e.g. distance from the village center; Fig. S4).

## Discussion

Understanding what drives infection heterogeneity is a key to predicting parasite transmission and spillover risk. However, identifying structured patterns in infection heterogeneity and linking them to underlying drivers remains challenging. By combining SBM and machine learning with detailed field data on the introduced *R. rattus* in Madagascar, we identified three distinct host infection profiles, ranging from no infection and weak protozoan associations to high infection richness dominated by a small number of highly prevalent taxa. Host traits were $\sim$40% more important than environmental factors in shaping these profiles, with body mass, gut microbiome composition, and small mammal community density emerging as the strongest predictors.

Hosts in profiles 2 and 3 differed not only in parasite richness but also in parasite composition, with a shift from highly prevalent *Tritrichomonas* OTUs in profile 3 to less prevalent *Hypotrichomonas* and other rare taxa in profile 2. The high prevalence of *Tritrichomonas* constitutes a dominant axis of infection heterogeneity. The profiles we detect, therefore, capture epidemiologically relevant differences among hosts: profile 3 hosts were primarily associated with generalist parasites and may act as superspreaders, whereas profile 2 hosts exhibited lower overall infection rates but harbored a more diverse assemblage of rarer taxa. Such contrasting infection signatures suggest that hosts occupying different profiles may play distinct roles in infection dynamics and spillover risk. Supporting this interpretation, profile 3 was more strongly linked to *Tritrichomonas*, while profile 2 was associated with *Blastocystis* and *Eimeria*. All three protozoan taxa were detected in local human populations [82], suggesting that the relevance of each profile to spillover events may differ .

Body mass was the dominant predictor of infection heterogeneity. Body mass is a well-established correlate of infection risk, often serving as a proxy for body condition, which can influence immune function and physiological resilience to parasites [59]. It also correlates with host age and sex, since younger and female rats tend to be smaller. Age influences infection patterns because younger individuals have had less time to accumulate parasites but may be more susceptible to initial infections [77], whereas male behavioral traits—greater movement, territoriality, and aggression—can increase exposure and infection rates [57]. Therefore, body mass likely integrates multiple underlying physiological processes (e.g. immune maturity and energy reserves) that drive parasite susceptibility. None of those traits emerged as top predictors on their own, possibly because their signal was outweighed by mass in the model. Future work that disentangles how age, sex, and body condition independently influence parasitism will help clarify which specific processes underlie the emergence of infection profiles.

Gut microbiome composition was also an important feature. While the microbiome is closely tied to host health, the direction of causality remains unclear: dysbiosis (an imbalance in microbial communities) may increase susceptibility, or alternatively, parasite infection itself can disrupt the microbiome and trigger dysbiosis [62, 83]. Moreover, microbiome composition shifts along environmental gradients such as land-use change, potentially influencing host–parasite dynamics [38, 84]. Therefore, regardless of causality, our findings highlight the importance of jointly considering microbiome and environmental factors, and suggest that the microbiome may serve as a useful indicator of infection heterogeneity in wild populations. Notably, unlike the microbiome, nematode co-infection did not emerge as an important predictor, although it is known to modulate host immunity and alter susceptibility to other parasites [85, 86]. More detailed research into how nematodes and protozoa jointly shape infection profiles is needed, particularly because their transmission pathways are similar.

The most influential environmental features were small mammal population densities. These community-level factors shaped infection profiles, particularly in the shift from no infection (profile 1) in natural habitats to higher infection rates (profile 3) in disturbed environments (e.g. villages, rice fields, and agroforests). Higher densities of rats and other non-native species were associated with these sites (Fig. S7), suggesting that increased host abundance and altered community composition facilitate parasite transmission. One mechanism is density dependence: denser populations lead to more frequent contact events and greater environmental contamination [40], especially for gut protozoa relying on fecal–oral transmission [44]. While vegetation and distance from villages were also included as proxies for land-use change, they were less predictive than small mammal density (Fig. S4). This suggests that land-use change influences infection primarily through its effects on host abundance and community composition [87].

Structured infection profiles both emerge from and reinforce eco-evolutionary feedbacks: host–parasite coevolution generates heterogeneity in infection patterns, which in turn biases transmission pathways and shapes subsequent evolutionary trajectories. Within this framework, infection profiles can be viewed as emergent ecological niches defined by characteristic combinations of hosts and parasites. SBM is well suited to capture these dynamics because it jointly classifies hosts and parasites based on their interaction patterns, naturally accounting for co-infection structure and heterogeneous prevalence. By revealing blocks that correspond to distinct host profiles and parasite niches, SBM links observed infection heterogeneity to underlying differences in interaction probabilities. Analyzing the host traits and environmental factors that determine membership in these profiles is therefore crucial, as it identifies the mechanisms that generate and maintain infection heterogeneity, clarifies how ecological context and host biology shape parasite niches, and provides insight into how eco-evolutionary feedbacks may respond to environmental change.

Our study advances understanding of how host traits and environmental variation interact to shape infection heterogeneity. Nevertheless, several limitations highlight directions for future research. First, we were unable to disentangle the relative contribution of host susceptibility from exposure [34]. Many predictors, such as gut microbiome composition, likely reflect both processes: the microbiome can influence immune function and infection risk [63, 88], but could also reflect shifts with diet and environment, serving as a proxy for exposure [84, 89]. Disentangling these processes will require experimental or longitudinal designs. Second, our model showed limited predictive performance, particularly for the low-infection host profile 2. This is likely in part due to class imbalance, as profile 2 comprised the smallest proportion of hosts (24.3%). Additionally, our model does not explicitly capture stochasticity in infection dynamics [90]. However, among the deterministic factors considered, we were able to distinguish those with greater predictive importance. Improving model performance and biological interpretability may require increased sampling effort and the inclusion of higher-resolution data—such as parasite load, behavioral metrics, and immunological markers.

In summary, our approach enables the detection of structured host–microbe associations and a mechanistic understanding of their emergence, bridging network analysis with predictive modeling in a way rarely applied in microbial ecology. Although broadly applicable to diverse microbial communities, our study system further highlights the interface between microbial and disease ecology. By integrating network-based approaches with machine learning and comprehensive ecological and biological data, we quantified the relative contributions of intrinsic host traits and extrinsic factors in shaping protozoan infection patterns of rats along a land-use gradient. This framework moves beyond traditional analyses by capturing complex co-infection dynamics and revealing the functional roles of different host groups in pathogen transmission. As anthropogenic disturbance continues to reshape host and microbial communities, such integrative approaches will be essential for advancing predictive microbial ecology, improving our understanding of community assembly processes, and informing strategies for infection risk assessment.

## Supplementary Material

ycag026_Supplemental_File

## Data Availability

Metadata and code have been uploaded to Zenodo (https://doi.org/10.5281/zenodo.18127198). Parasite sequence data are available in the NCBI Sequence Read Archive under accession PRJNA1400020. Microbiome sequence data are available in the NCBI Sequence Read Archive under accession PRJNA1345735. Data and code are also available on the GitHub repository https://github.com/MadagascarEEID/rat_protozoa_infection_profiles.
