# Supplementary material for: Host traits and environmental factors shape infection heterogeneity in wild rat–protozoa networks
Source: ISME Commun. 2026 Feb 10;6(1):ycag026. doi: 10.1093/ismeco/ycag026 (PMC12930476; doi:10.1093/ismeco/ycag026)
Supplement: ycag026_Supplemental_File [file ycag026_supplemental_file.pdf]

Supplementary information: Host traits and environmental factors shape infection heterogeneity in wild rat–protozoa networks

Matan Markfeld [1,†], Itamar Talpaz [1,†], Barry Biton [1], Toky Maheriniaina Randriamoria[2], Voahangy Soarimalala [2,3], Steven M. Goodman [2,4], Charles L. Nunn [5,6], Georgia Titcomb [7,*], and Shai Pilosof [1,8,*]

[1]Department of Life Sciences, Ben-Gurion University of the Negev, Be'er-Sheva, Israel
[2]Association Vahatra, Antananarivo, Madagascar
[3]Institut des Sciences et Techniques de l'Environnement, Université de Fianarantsoa, Fianarantsoa, Madagascar
[4]Field Museum of Natural History, Chicago, IL, USA
[5]Department of Evolutionary Anthropology, Duke University, Durham, NC, USA
[6]Duke Global Health Institute, Durham, NC, USA
[7]Department of Fish, Wildlife, and Conservation Biology, Colorado State University, Fort Collins, CO, USA
[8]The Goldman Sonnenfeldt School of Sustainability and Climate Change, Ben-Gurion University of the Negev, Be'er Sheva, Israel
[†]Equal contribution
[*]Corresponding authors: pilos@bgu.ac.il, georgia.titcomb@colostate.edu

Table of contents:

- Table S1: Summary of features used in the XGBoost model
- Table S2: Hyperparameter grid search settings for the XGBoost model
- Figures S1-4
- SI note 1: Study site and small mammal sampling
- SI note 2: Measuring host traits and environmental features
- SI note 3: Details on model evaluation metrics
- SI note 4: Results of model evaluation
- References

Table S1: Summary of features used in the XGBoost model. See **SI note 2** for detailed explanations on sampling and measurement methods.

Category	Sub-category	Feature	Type	Scale	Explanation	References
Host traits	Biological	Body mass	Continuous	Individual	Rat body mass [gram]	[1,2]
		Body condition	Continuous	Individual	Body Condition Index value by age and sex	
		Sex	Binary	Individual	Male / female	[3,4]
		Age	Binary	Individual	Sub-adult / adult	[5–7]
	Nematode co-infection	Nematode co-infection	Binary	Individual	Infection by any nematode species [0/1]	[8–10]
	Microbiome	*Lachnospiraceae*	Continuous	Individual	Family relative abundance [0-1]	[11–14]
		Lactobacillaceae	Continuous	Individual	Family relative abundance [0-1]	
		Muribaculaceae	Continuous	Individual	Family relative abundance [0-1]	
		Prevotellaceae	Continuous	Individual	Family relative abundance [0-1]	
Environmental	Vegetation	Vegetation PC1	Continuous	Site	Habitat attributes PC1	[15–17]
		Vegetation PC2	Continuous	Site	Habitat attributes PC2	
	Distance	Village distance	Continuous	Individual	Distance in [m] from the nearest village center	
	Community density	Rat density	Continuous	Site	Density of rat population at the site	[18–21]
		Non-native density	Continuous	Site	Density of non-native (shrews and house mice) at the site	
		Native density	Continuous	Site	Density of native sp. populations at the site	

Table S2: Hyperparameter grid search settings for the XGBoost model.

Hyperparameter	Values	Description
max_depth	1, 2, 3	Maximum depth of a tree; controls model complexity.
eta	0.002, 0.01, 0.1	Learning rate; scales the contribution of each tree.
colsample_bytree	0.4, 0.6	Fraction of features sampled per tree.
min_child_weight	6, 8	Minimum sum of instance weight needed in a child.
subsample	0.5, 0.7	Fraction of training data sampled per tree.
lambda	5, 10	L2 regularization term on weights; reduces overfitting.
alpha	2, 5	L1 regularization term on weights; encourages sparsity.

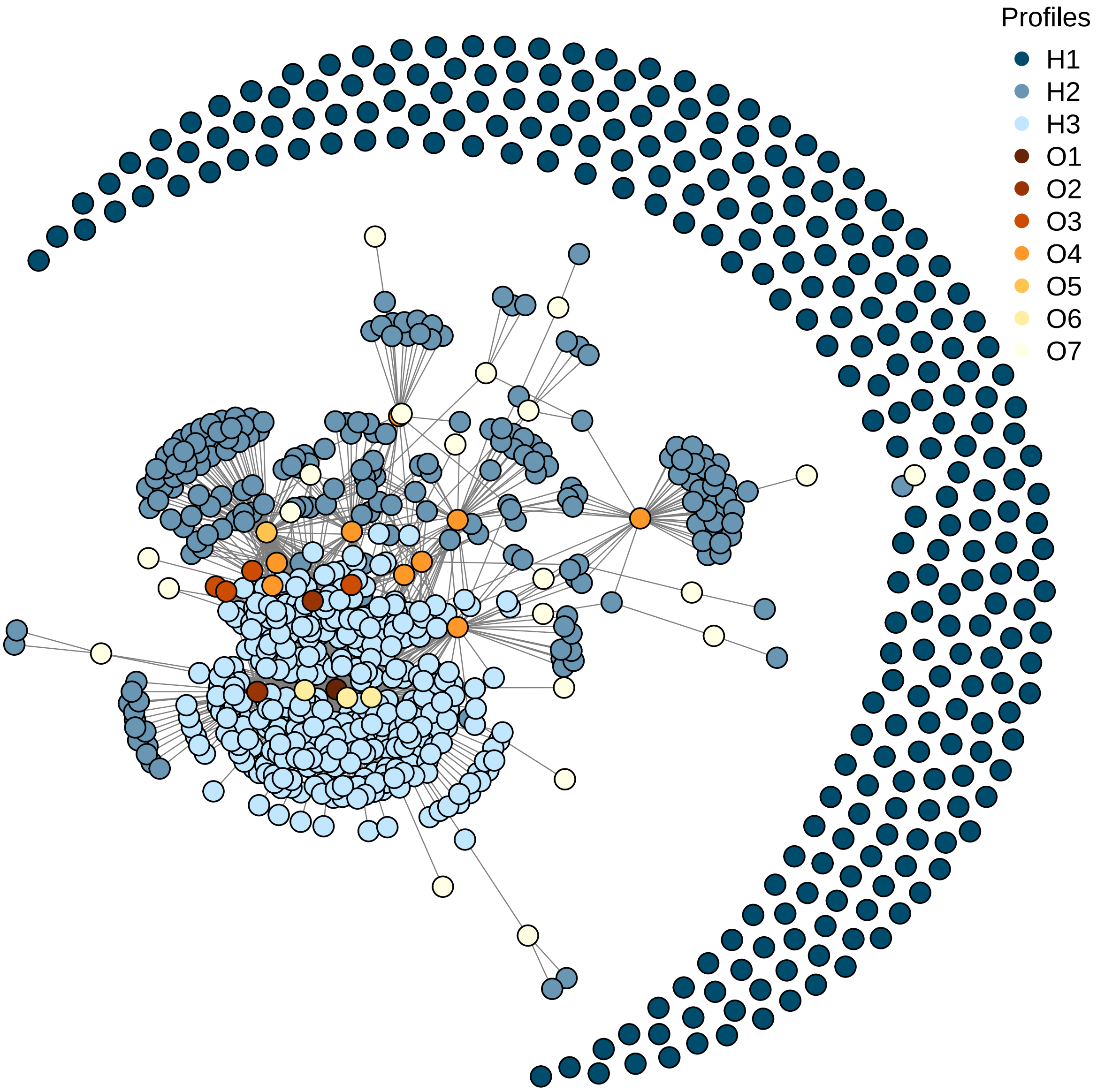

Figure S1: Visualization of the rat-protozoa bipartite network. Nodes represent individual rat *Rattus rattus* hosts and protozoa OTUs, with edges indicating infections. Blue-shaded nodes denote host infection profiles, while yellow-to-red shaded nodes represent protozoa infection profiles.

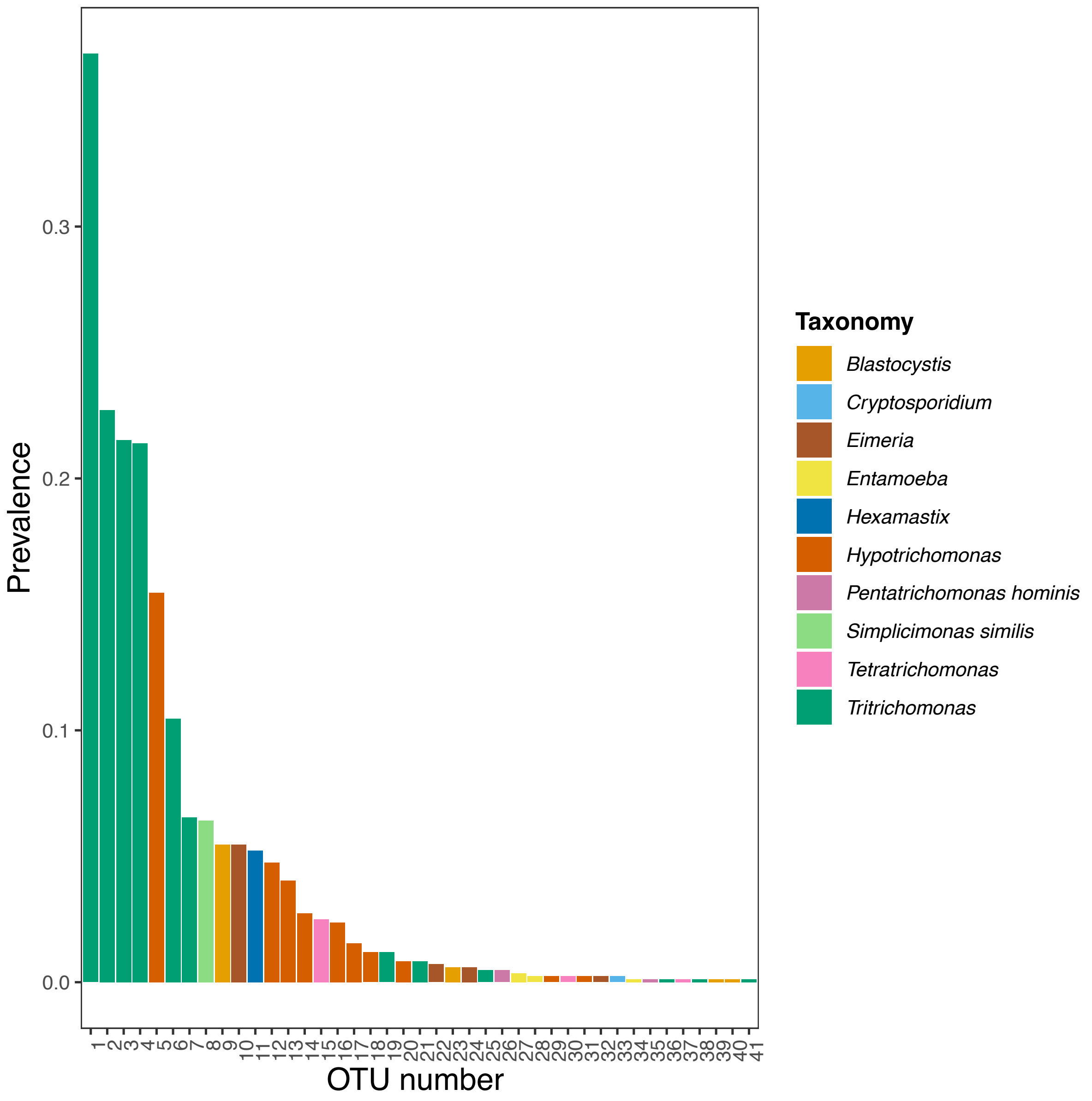

Figure S2: Protozoa OTUs prevalence distribution and taxonomy. The prevalence of 41 OTUs, measured as their occupancy out of the 841 hosts, ordered from highest to lowest. Colors represent the lowest taxonomic classification (genus or species) of each OTU.

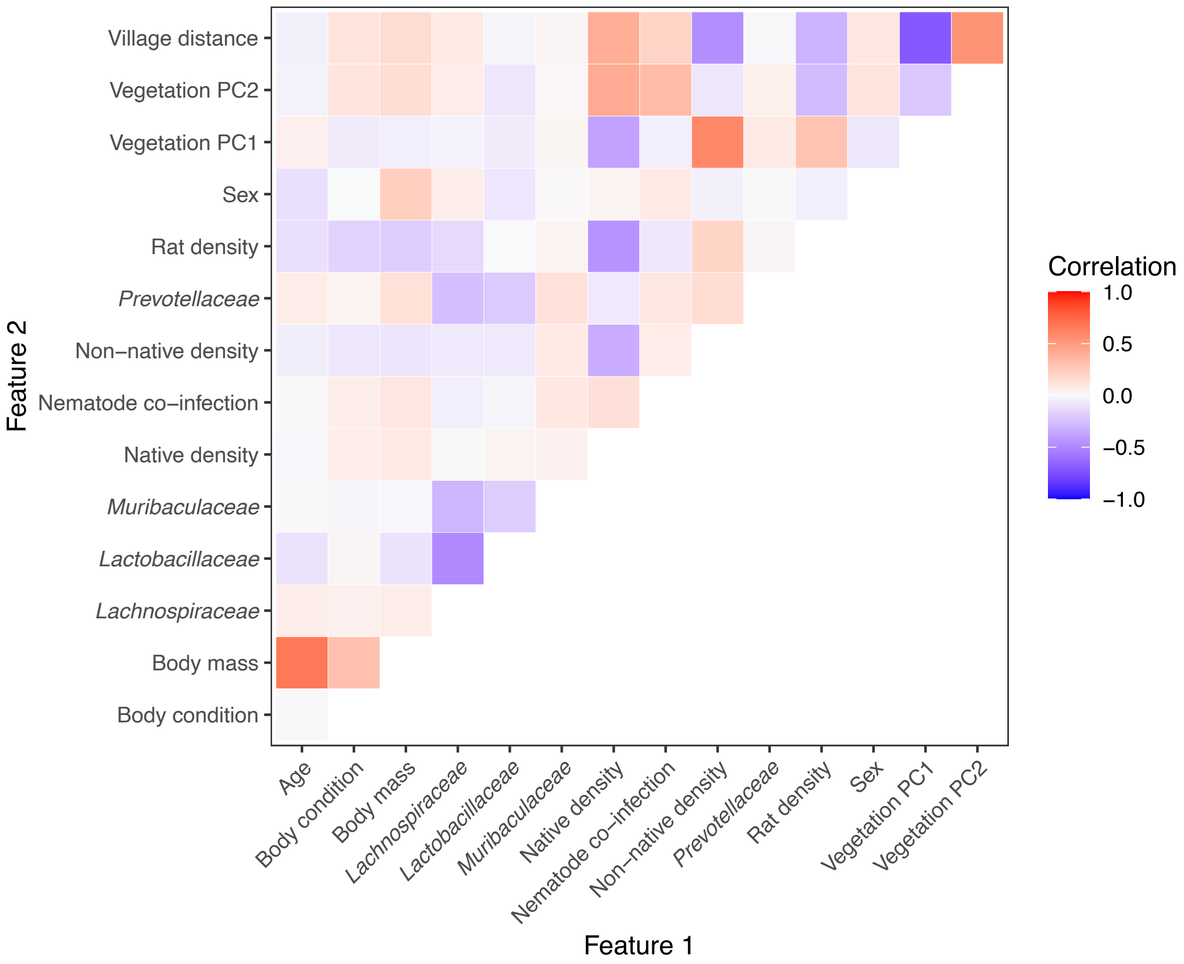

Figure S3: Correlations between features. Pairwise correlations were computed between all features. Pearson correlation was used for continuous–continuous pairs, point-biserial correlation for continuous–dichotomous pairs, and tetrachoric correlation when both features were dichotomous. Colors represent the strength and direction of the correlations: red indicates positive, and blue indicates negative relationships.

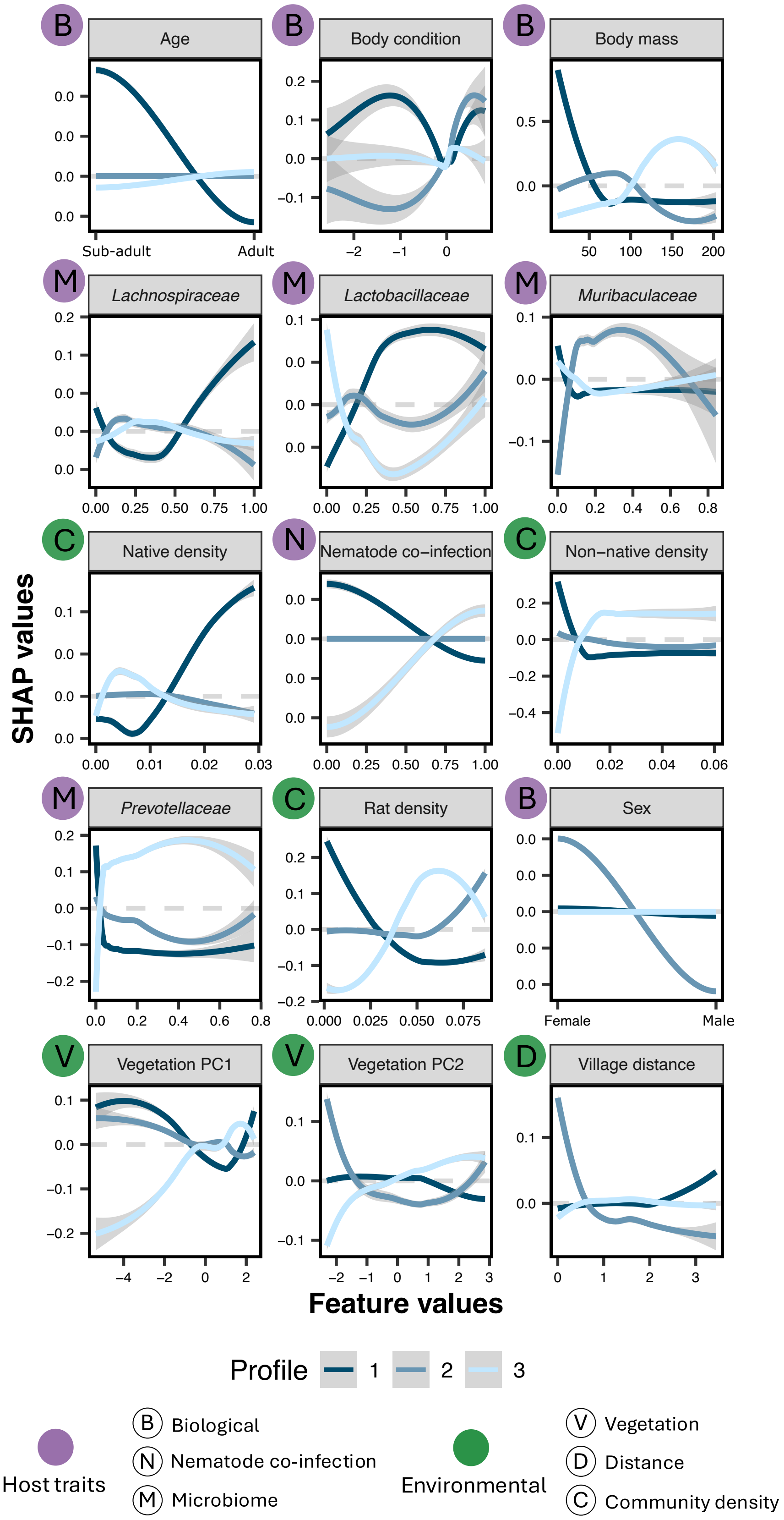

Figure S4: SHAP dependency plots for all features. SHAP dependency plots show individual hosts' SHAP values as a function of feature values. For clarity, we visualized only the LOESS-derived trendline and its associated confidence interval for the 841 host samples, rather than displaying all individual host data points. Line colors represent different host infection profiles, while the dashed gray line marks a SHAP value of zero. Y-axis values were rounded to two decimal places.

SI note 1: Study site and small mammal sampling

Small mammals were collected in the vicinity of three villages in the SAVA Region of northeast Madagascar, in the surroundings of Marojejy National Park. The village of Mandena (14.477049° S, 49.8147° E) was sampled between October 2019 and September 2020. A second village, Sarahandrano (14.607567° S, 49.647759° E), was sampled between November 2020 and September 2021, while a third village, Andatsakala (14.397276° S, 49.8820° E), was sampled between October 2021 and August 2022. In the vicinity of each village, seven sites were sampled along a degradation gradient: (1) semi-intact natural forest inside the national park, (2) secondary forest, (3) *savoka* (brushy regrowth), (4) agroforest (vanilla plantation), (5) mixed agriculture (sugarcane/coffee plantation), (6) flooded rice, and (7) the village itself. Sites near each village were located ∼500 m apart.

For sampling small mammals, a 100 m X 100 m grid of 121 live traps (11x11) was established, including 97 Sherman (H. B. Sherman Traps, Inc., Tallahassee, Florida, model LFA and XLK), and 24 Tomahawk (Tomahawk Live Trap, Hazelhurst, Wisconsin, model 201), placed 10 m apart and baited with peanut butter. Additionally, two pitfall lines were installed between 20-50 m outside of the grid, running in parallel to the grid edge. Each pitfall line was 100 m in length, with 11 buckets dug into the ground and placed every 10 m, and an 80 cm high vertically oriented plastic fencing bisecting each bucket, stapled to vertical stakes, and a flange touching the ground covered with soil and leaf litter to block the passage of small mammals and guide them to a bucket. Each plot was sampled for six consecutive nights and during three different sampling periods (before the wet season, after the wet season, and during the dry season).

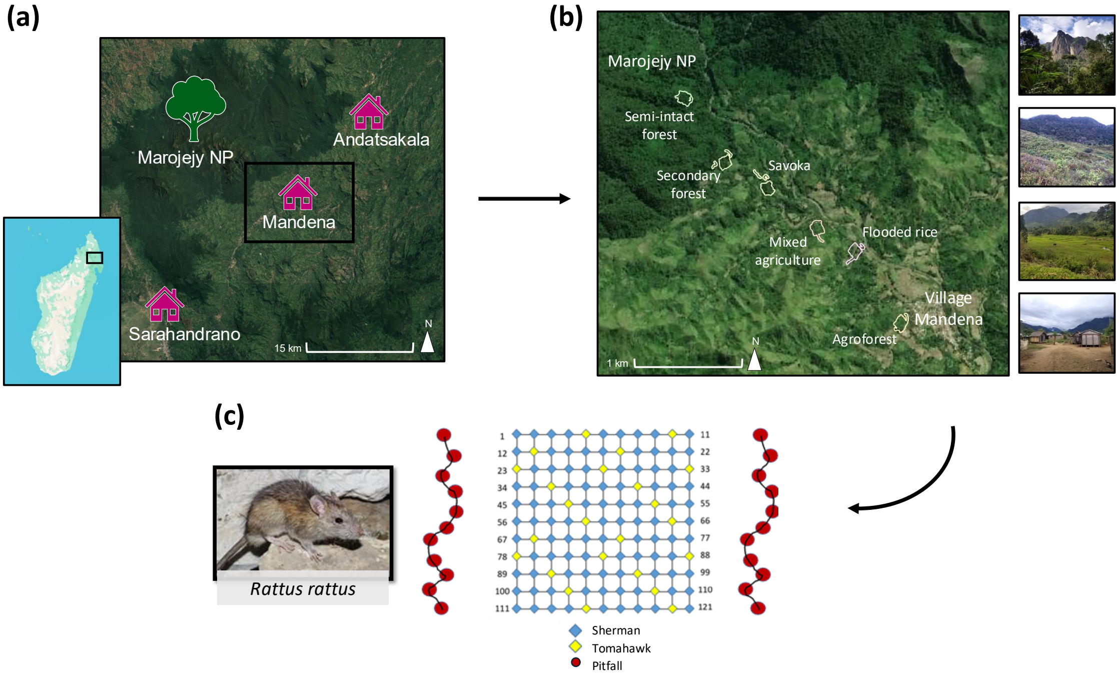

Figure S5: Study site and sampling scheme. **(a)** Sampling was conducted in northeastern Madagascar, in three different zones associated with three villages near Marojejy National Park. **(b)** In each village, seven distinct land-use types were sampled. The map illustrates the village of Mandena as an example. The images depict typical landscapes from top to bottom: semi-intact forest, *savoka*, flooded rice fields, and village plots. **(c)** In each plot, an 11×11 trapping grid consisting of Sherman and Tomahawk traps was installed, along with two pitfall lines. A total of 841 individual *Rattus rattus* were captured.

SI note 2: Measuring host traits and environmental features

To explore determinants of parasite infection patterns, we measured three environmental variables (vegetation, small mammal community, and distance to the village center) and six host variables (mass, body condition index, sex, age, nematode co-infection, and gut microbiome composition) (**Table S1**). The host variables and distance to the village are specific to each individual rat, while the environmental variables are specific to a site and common to all the rats captured at a specific site in a specific season.

Habitat attributes

We measured two environmental gradients across sites: vegetation and the distance from the village center. These variables collectively capture much of the natural and anthropogenic variation across the landscape and are related to the environmental reservoir of parasites. The distance to the nearest village was measured using a GPS logger as the shortest distance from the village center to the trap location where the rat was captured.

In addition, with the help of a specialist botanist, we measured habitat attributes in 16 plots (5m $\times$ 5m) within the sampling grid at each site, conducting measurements three times (seasons) during the sampling period. At each plot, we assessed eight habitat characteristics: (1) number of trees, (2) number of dead logs, (3) tree diameter at breast height, (4) tree height, (5) percent canopy cover, (6) number of lianas, (7) herbaceous vegetation height, and (8) percent herbaceous vegetation cover. We averaged the measurements across all plots to calculate mean values for each site per season. To explore habitat variation between sites, we conducted a principal component analysis (PCA). Prior to analysis, all variables were centered at 0 and rescaled to have unit variance. The first two principal components explained 80.71% of the variation across sites (PC1: 59.51%, PC2: 21.2%) (**Figure S6**). Vegetation PC1 divides the more natural sites (semi-intact forest and secondary forest) from the more disturbed sites. PC2 is positively correlated with herbaceous vegetation cover and height.

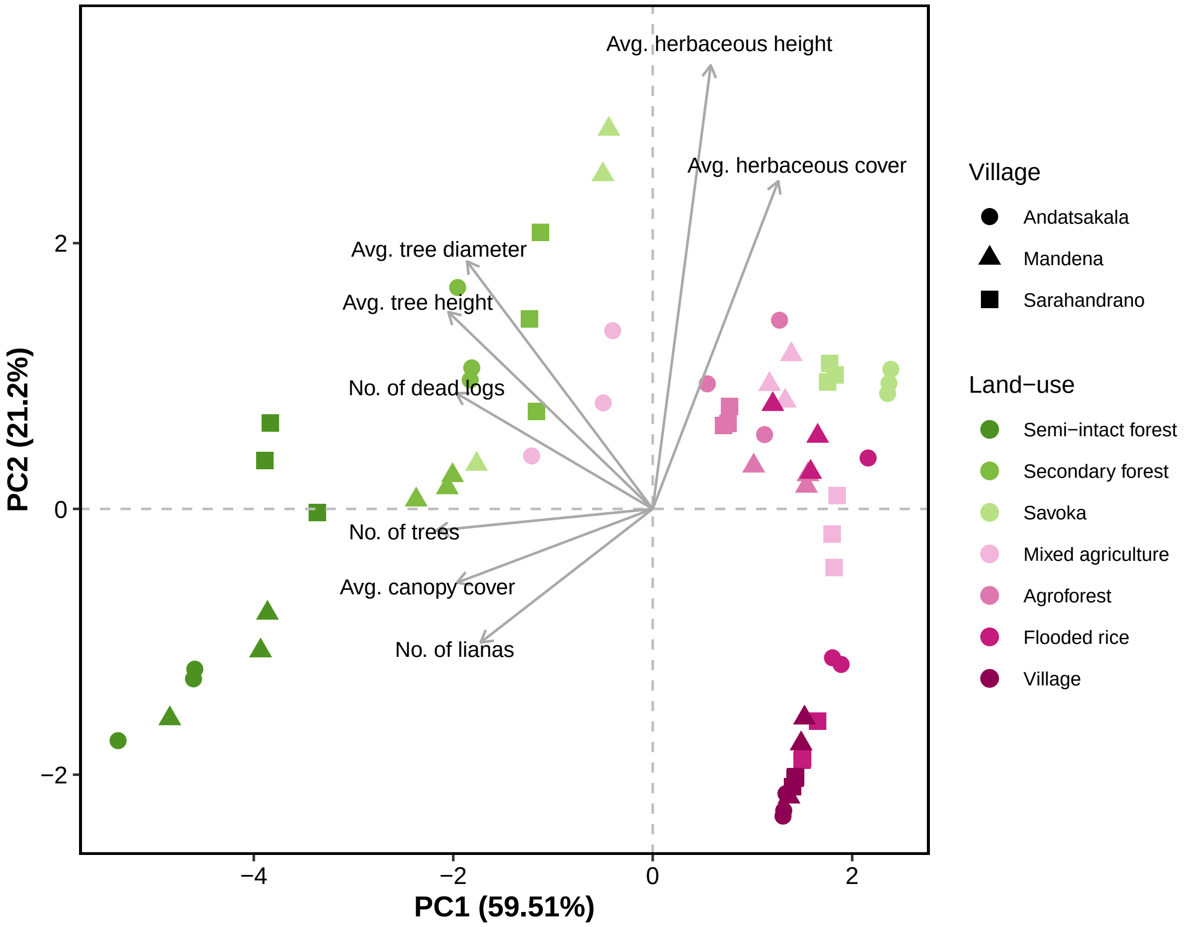

Figure S6: Vegetation PCA across land-use types. The first two principal components (PC1 and PC2) from the PCA of vegetation attributes. Each point represents a site in one season, with shape denoting the village and color representing the land-use type. Arrow length and direction indicate the contribution of each vegetation variable to the first two PCs.

Small mammal community

Population density can influence infection patterns, as higher contact rates in denser populations may increase parasite transmission within and between host species. Therefore, for each site and season, we measured small mammal density for (1) the rat (*Rattus rattus*) population, (2) other non-native species (including *Mus musculus* and *Suncus* spp.), and (3) native species (including members of the family Tenrecidae and subfamily Nesomyinae). Density was calculated as the total abundance of individuals in categories 1–3 at a given site-season, divided by the number of traps at that site. For the rat population, pitfall traps were excluded from calculations, as

only two individuals (0.2%) were captured in them. The small mammal community significantly differed between more natural sites (semi-intact forest and secondary forest) and more disturbed sites (**Figure** S7).

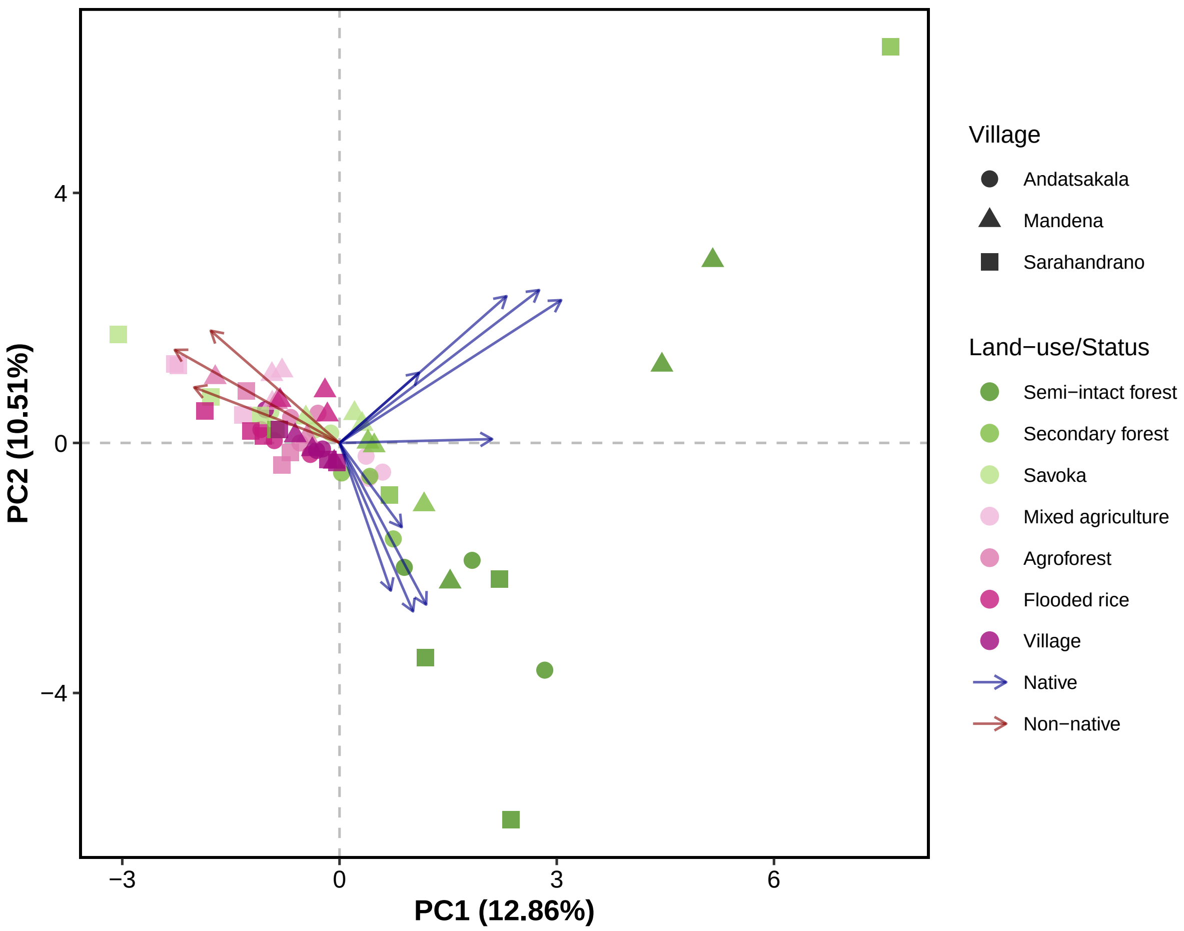

Figure S7: Small mammal community PCA across land-use types. The first two principal components (PC1 and PC2) from the PCA of small mammals species. Each point represents a site in one season, with shape denoting the village and color representing the land-use type. Arrow length and direction indicate the contribution of each species to the first two PCs. The arrow colors indicate native (blue) and non-native (red) species.

Body condition

To assess the physiological condition of individual rats, we calculated a Body Condition Index (BCI) based on the residuals from a linear regression of body mass on structural body length. Because body size and growth patterns can differ significantly between age classes and sexes,

we calculated BCI separately for each combination of age group (sub-adult/adult) and sex. For each subgroup, we log-transformed both body mass (M) and head-body length (L) to linearize the allometric relationship. We then fit a linear model of the form:

$$\log(M_i) = \beta_0 + \beta_1 \log(L_i) + \varepsilon_i \tag{S1}$$

where M_i is the mass of individual i, L_i is its head-body length, β_0 and β_1 are the intercept and slope of the regression, and ε_i is the residual. The residuals ε_i from this regression represent the BCI, with positive values indicating individuals heavier than expected for their body length (i.e., better condition), and negative values indicating poorer condition. These residuals were used as a continuous predictor of host condition in the subsequent statistical model.

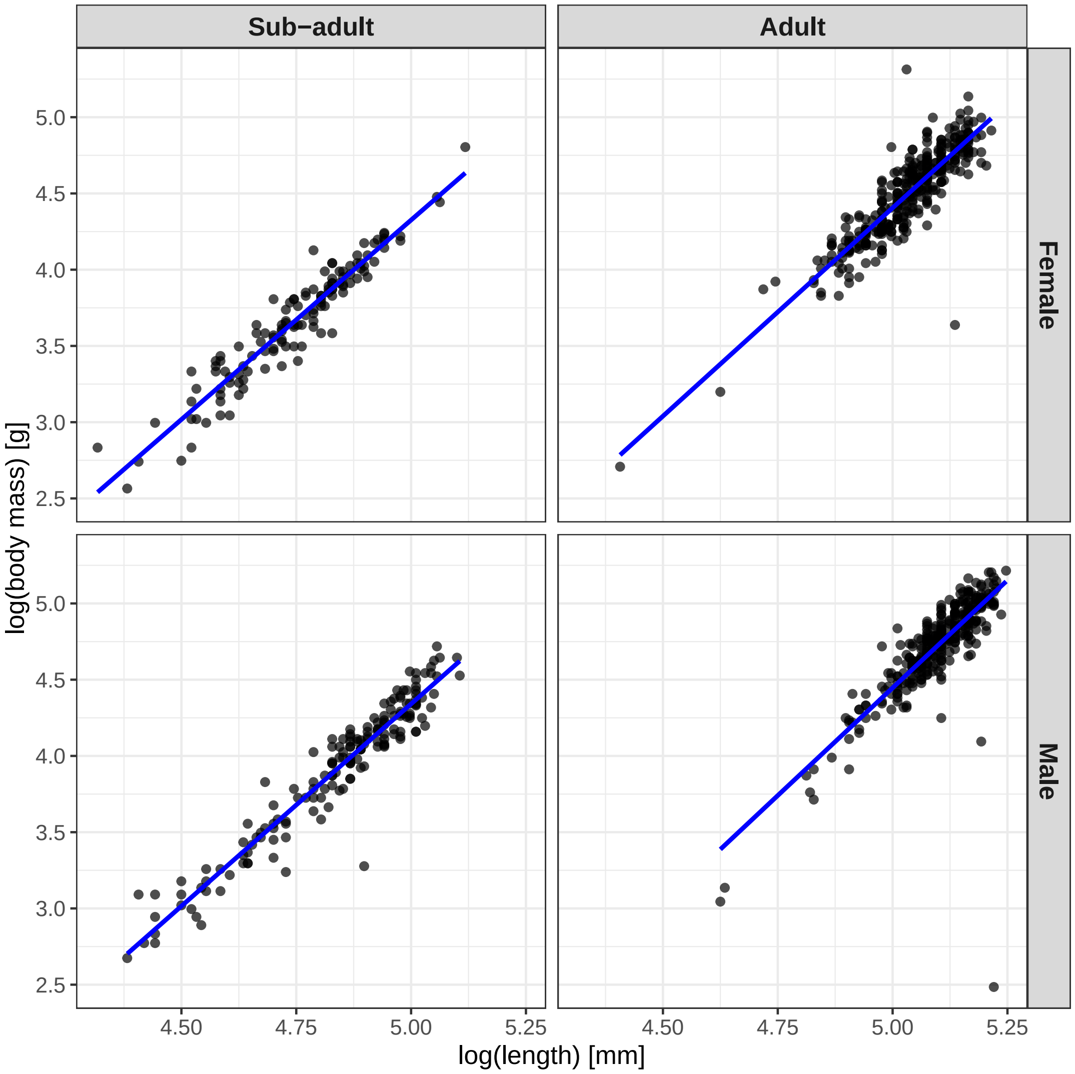

Figure S8: Body Condition Index (BCI) of individual rat hosts. BCI was calculated by the residuals from a linear regression of body mass [g] on structural body length [mm] for each combination of age group (sub-adult/adult) and sex (female/male).

Gut microbiome

DNA was extracted from ~1g feces collected from trapped small mammals using Zymo Quick-DNA Fecal/Soil Microbe Miniprep kits (cat #D6010) using manufacturer protocols. 16S metabarcoding was conducted using 515F–806R primers to target the V4 region of the 16S SSU rRNA [22]. Each primer included an Illumina adapter, barcode, primer pad, and linker. Reactions were carried out in 25 μL volumes consisting of 10 μL of 1.25 μM forward and reverse primer, 2 μL of DNA, and 13 μL of Platinum Hot Start PCR mastermix (ThermoFisher Scientific, cat #13000014). Reaction conditions were as follows: 95°C for 3min, 35x 98°C for 30secs, 58°C for 30secs, and 72°C for 30 s, followed by a final extension at 72°C for 5min. Concentrations were measured using Promega One Quantifluor kits on a Tecan plate reader. Samples were then normalized to 7 ng/μL prior to pooling. The product was cleaned using magnetic beads (bead:DNA ratio was 0.8:1) and sequenced at UC Santa Barbara Biological Nanostructures Laboratory on an Illumina MiSeq (v3 chemistry, 2x300 bp, 24M reads).

Sequences were demultiplexed using cutadapt (v.3.4) with zero error tolerance [23]. We then performed quality filtering steps using the *dada2* package in R [24]. Specifically, we filtered and trimmed amplicons (minimum length = 100, 15% PhiX removed), inferred and removed errors, dereplicated sequences, inferred amplicon sequencing variants (ASVs) using the pseudo-pooling method, merged pairs, and removed chimeras. We assigned taxonomic identifications to ASVs using the *assignTaxonomy* function in *dada2*, using the SILVA nr99 SSU reference database (v.138.1).

We filtered out very rare ASVs with a relative abundance lower than 0.1% in a sample or those that occur in less than 1% of all individuals. Additionally, we removed all non-bacterial ASVs or those that were identified as 'Chloroplast' or 'Mitochondria'. Finally, we excluded 21 samples with fewer than 5000 total reads from our analysis. Filtering procedures resulted in 1,951 ASVs from an original total of 10,358.

We aggregated ASVs at the family level for each individual host. ASVs with unidentified families were excluded, resulting in the analysis of 1,770 ASVs (90.72% of all ASVs) classified into 55 families. To examine microbiome variation among individuals, we performed a principal coordinate analysis (PCoA). The first two principal coordinates explained 56.8% of the variation (PCo1: 36.5%, PCo2: 20.3%) (**Figure S9**). For better interpretability, we selected as features only the microbial families that exhibited a significant correlation with the first two principal coordinates (PCos). To achieve this, we utilized the equilibrium circle (or correlation circle), a graphical tool that helps interpret the contribution of variables to the principal coordinates.

The radius of the equilibrium circle is given by Radius $\propto \left(\frac{d}{p}\right)^{0.5}$, where d represents the number of retained principal coordinates (two in our case), and p denotes the total number of original variables. A variable (microbial family) with a vector extending beyond the equilibrium circle indicates a strong correlation with at least one principal coordinate, signifying a major role in the ordination. Conversely, vectors within the circle reflect weaker correlations and lower contributions to the PCoA structure. Based on this criterion, we identified four microbial families as significant and included them as features in the final XGBoost model: (1) *Lachnospiraceae*, (2) *Lactobacillaceae*, (3) *Muribaculaceae*, and (4) *Prevotellaceae*.

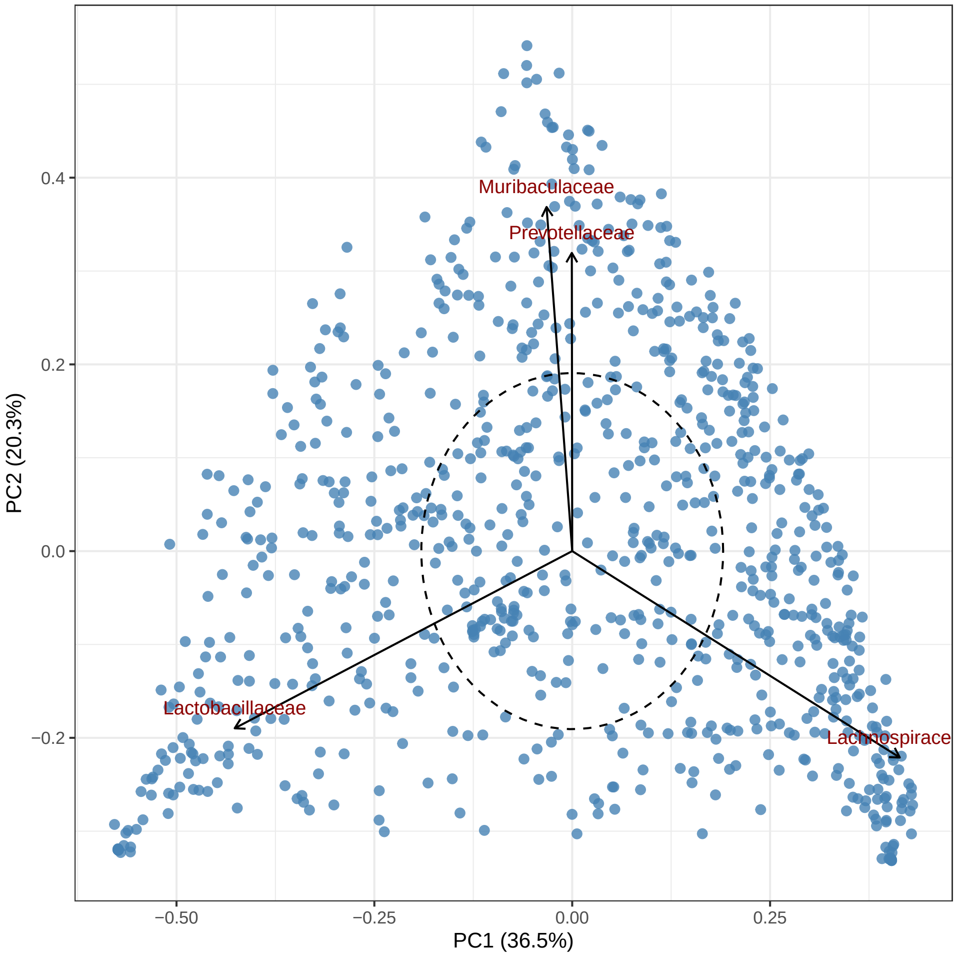

Figure S9: PCoA of the gut microbiome across individual rat hosts. The first two principal coordinates (PCo1 and PCo2) are shown. Each point represents an individual rat, with the circle marking the equilibrium threshold. Arrow length and direction indicate the contribution of each microbial family to the first two PCos, with only families whose arrows extend beyond the equilibrium circle displayed.

Nematode co-infection

We performed metabarcoding using the NC1/NC2 primer set [25] to amplify ITS2 ribosomal DNA from strongylid nematodes. Forward and reverse primers contained 8-nucleotide barcodes

with a Hamming distance of at least 4. PCR reactions were carried out in 15μL volumes consisting of: 3 μL of each forward and reverse primer (2 μM stock concentration); 7 μL from a Mastermix comprised of 0.7 μL of Amplitaq Gold polymerase, 150 μL MgCl2, 150 μL Amplitaq Gold buffer, 12 μL BSA, 6 μL DMSO, and 344 μL water; and up to 2 μL template DNA (1–100 ng total). Cycling conditions were: 10-minute hot-start activation, 35x cycles of 15 s at 95°C, 30 s at 55°C, 40 s at 72°C, and a final 5-min extension at 72°C. DNA concentrations were then measured, pooled, normalized, and purified using MinElute columns prior to multiplexing with additional libraries. The final library for each village was sequenced three times on an Illumina MiSeq (v3 2 × 300 bp, 25 M reads) at the UC Davis Genome Center. Sequences were demultiplexed using cutadapt (v.3.4) with zero error tolerance [23]. We used the *dada2* bioinformatics pipeline [24] to filter and trim amplicons (minimum length = 100, 15% PhiX removed), remove errors, dereplicate, infer amplicon sequence variants (ASVs) using the pseudo-pooling method, merge pairs, remove chimeras, and combine the three ASV read tables from the different villages into one table. We then calculated the relative read abundance of each ASV and excluded reads that accounted for less than 1% of a sample's relative read abundance to avoid potential sequencing errors or tag jumps. We excluded a small subset of samples that failed to amplify across other primer sets, and used the assignTaxonomy function with minimum bootstraps = 50 to identify ASV sequences using the nemabiome ITS2 reference database (v 1.6.0). Next, we clustered phylogenetically similar ASVs into OTUs at 97% similarity using the 'Clusterized' function from the *DECIPHER* package. Taxonomy was assigned to each OTU based on its most common ASV.

To examine variation in nematode co-infection among individual hosts, we plotted the distribution of the number of nematode OTUs infecting each host (**Figure S10**). Because the distribution was highly skewed, with most rats infected by only a single nematode OTU, we included a binary variable in the final model indicating whether or not the host was infected by any nematode species (infected: n = 626; uninfected: n = 215).

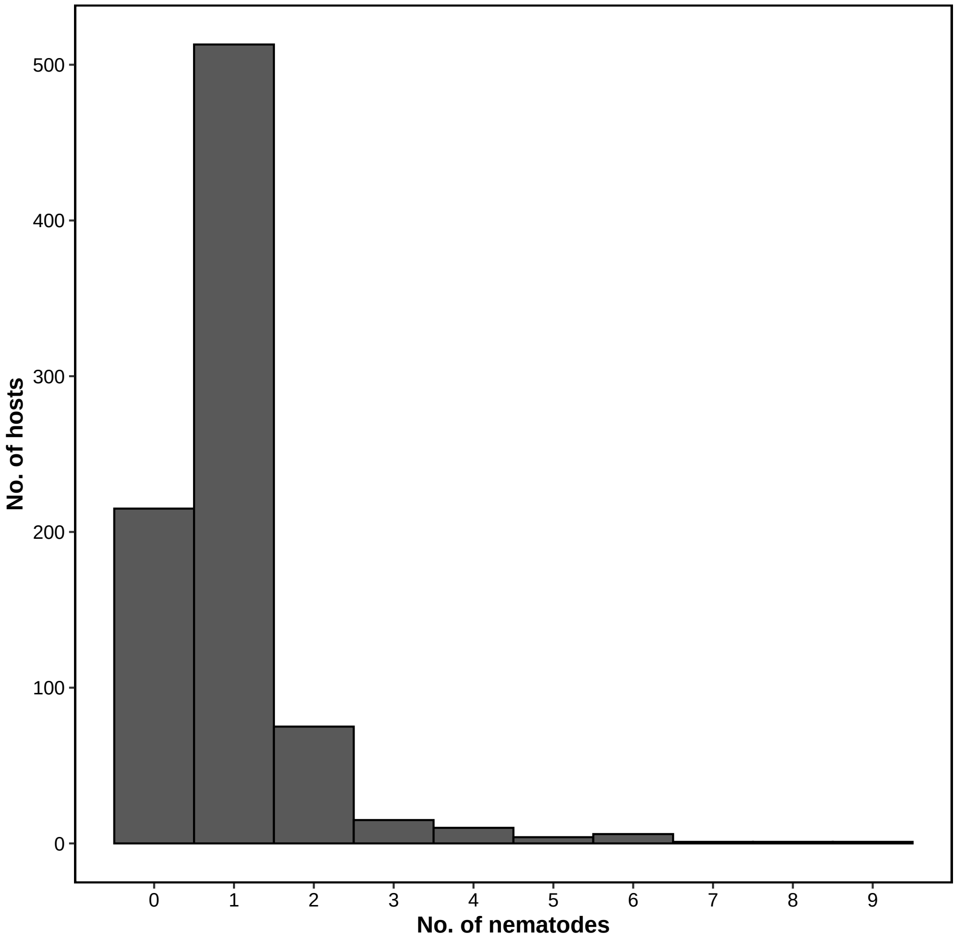

Figure S10: Distribution of the number of nematode OTUs infecting each host.

SI note 3: Details on model evaluation metrics

In multi-class classification models, the output is a probability distribution over the possible classes (i.e., the probabilities of a host being classified into one of the host infection profiles). This is achieved using a softmax function, which converts raw scores into probabilities that sum to 1. The predicted class is the one with the highest probability.

To evaluate the performance of our three-class classification model, we used a confusion matrix, which records the number of correctly and incorrectly classified instances for each class [26]. The confusion matrix is structured as follows:

Table S3: Confusion Matrix for a 3-Class Model. A-C are host infection profiles.

Actual \ Predicted	Pred A	Pred B	Pred C
**Actual A**	TP_A	$FP_{B,A}$	$FP_{C,A}$
**Actual B**	$FP_{A,B}$	TP_B	$FP_{C,B}$
**Actual C**	$FP_{A,C}$	$FP_{B,C}$	TP_C

Each row represents the actual class, while each column represents the predicted class.

- TP_X (True Positives): Correctly classified instances of class X.

- $FP_{Y,X}$ (False Positives): Instances incorrectly classified as class Y when they actually belong to class X.

Since our dataset is imbalanced, we used evaluation metrics that give fair importance to each class based on the class size (i.e., fraction of hosts with the infection profile). These include accuracy, weighted precision, weighted recall, weighted F1-score, weighted balanced accuracy, and the Matthews Correlation Coefficient (MCC) [27].

Accuracy: Accuracy measures the overall correctness of the model:

$$\text{Accuracy} = \frac{TP_A + TP_B + TP_C}{N} \tag{S2}$$

where N is the total number of samples.

Weighted Precision: Precision for class X is the proportion of correctly predicted X instances out of all instances predicted as X:

$$P_X = \frac{TP_X}{TP_X + \sum FP_{X,Y}} \tag{S3}$$

The weighted precision is:

$$P_w = \sum_{X \in \{A,B,C\}} w_X P_X \tag{S4}$$

where w_X is the proportion of actual instances of class X.

Weighted Recall: Recall (Sensitivity) for class X measures how many actual X instances were correctly classified:

$$R_X = \frac{TP_X}{TP_X + \sum FP_{Y,X}} \tag{S5}$$

The weighted recall is:

$$R_w = \sum_{X \in \{A,B,C\}} w_X R_X \tag{S6}$$

Weighted F1-score: F1-score is the harmonic mean of precision and recall for each class:

$$F1_X = 2 \times \frac{precision \times recall}{precision + recall} \tag{S7}$$

The weighted F1-score is:

$$F1_w = \sum_{X \in \{A,B,C\}} w_X F1_X \tag{S8}$$

Weighted Balanced Accuracy: Balanced accuracy accounts for class imbalance and is calculated as the mean recall across classes:

$$BA_X = \frac{TP_X}{TP_X + \sum FP_{Y,X}} \tag{S9}$$

The weighted balanced accuracy is:

$$WBA = \sum_{X \in \{A,B,C\}} w_X B_X \tag{S10}$$

Matthews Correlation Coefficient (MCC): MCC is a more comprehensive metric that considers all values in the confusion matrix, and is, therefore, a balanced measure that can be used even if the classes are of very different sizes:

$$MCC = \frac{c \times s - \sum_{X \in \{A,B,C\}} p_X \times t_X}{\sqrt{\left(s^2 - \sum_{X \in \{A,B,C\}} p_X^2\right)\left(s^2 - \sum_{X \in \{A,B,C\}} t_X^2\right)}} \tag{S11}$$

where:

c = sum of true positives across all classes

s = total number of samples

p_X = predicted counts for each class $TP_X + FP_{XY}$

t_X = actual counts for each class $TP_X + FP_{YX}$

MCC values range from -1 to $+1$. A coefficient of $+1$ indicates a perfect prediction, 0 indicates no better than a random prediction, and -1 indicates total disagreement between prediction and observation.

To further assess model performance, we compared all metrics against a theoretical no-skill classifier, whose expected values were analytically computed based on profile distributions using proportional guessing. In this approach, the classifier predicts each profile according to its prevalence in the dataset, favoring frequent profiles over rare ones. This reflects the natural class distribution without relying on learned patterns. For this classifier, accuracy, precision, and recall scale with profile frequencies, while balanced accuracy remains equivalent to a uniform guessing strategy, providing a simple baseline despite class imbalance. The theoretical MCC for an ideal random classifier is zero. This method offers a straightforward way to benchmark our trained model's performance against random chance, especially in datasets with imbalanced classes.

In addition, we used evaluation metrics in a one-vs-all manner, where the predicted class is considered positive, and the remaining classes are treated as negative. The model's performance was then evaluated across multiple threshold values (i.e., classifying a sample into class X only if its probability exceeds threshold Y) by computing the Area Under the Receiver Operating Characteristic curve (AUC-ROC) and the Area Under the Precision-Recall Curve (PR-AUC) for each class separately.

ROC-AUC: The area under the receiver operating characteristic curve is a graphical representation of the actual positive rate (y-axis) versus the false positive rate (x-axis) of a model across different decision thresholds. The ROC-AUC score ranges from 0 to 1, where a score of 1 represents a perfect classification model, while a score of 0.5 represents a one-vs-all model with random guessing.

Although the ROC-AUC is a common measure, the number of true negatives in imbalanced data sets is very large, so even with a substantial number of false positives, the false positive rate might remain relatively small. This means that the ROC curve might not fully capture the cost of misclassifying a substantial number of the minority class instances. A better way to evaluate predictions in imbalanced data sets is by combining precision and recall metrics. Precision and recall provide a more granular understanding of a model's performance because their trade-off highlights how well the model balances false positives and false negatives, offering insight into its effectiveness in identifying true cases under different thresholds.

PR AUC: To evaluate the tradeoff between precision and recall, the area under the PR curve provides a single number that summarizes the overall performance of a model across all possible classification thresholds. Like the ROC-AUC curve, the PR curve is calculated across all thresholds. We calculated a PR curve for each class. The no-skill baseline PR-AUC was derived from class prevalence, meaning that a random classifier's expected precision equaled the fraction of positive instances in the dataset (i.e., the fraction of hosts in the infection profile out of all hosts).

SI note 4: Results of model evaluation

Our XGBoost model demonstrated strong overall performance, significantly predicting infection across all host profiles (**Figure S11**). All evaluation metrics exceeded those of a no-skill classifier, including accuracy (0.54), weighted precision (0.53), weighted recall (0.54), weighted F1-score (0.53), weighted balanced accuracy (WBA) (0.64), and Matthews correlation coefficient (MCC) (0.28) (**Figure S11A**).

The Area Under the Receiver Operating Characteristic Curve (AUC-ROC) was above the random value of 0.5 for all profiles (AUC = 0.726, 0.606, 0.738, for profiles 1, 2, and 3, respectively), indicating effective classification accuracy (**Figure S11B**). However, the relatively lower ROC curve for profile 2 (reflecting a low true positive to false positive rate) suggests that the model only weakly distinguished it from other profiles. The Precision-Recall Curve (PR-AUC) is useful for imbalanced datasets, as it captures the tradeoff between precision and recall. The PR-AUC was above the no-skill values (i.e., the fraction of hosts with each infection profile) for the three host profiles: AUC = 0.548 (no-skill of 0.322) for profile 1; AUC = 0.331 (no-skill of 0.244) for profile 2; and AUC = 0.656 (no-skill of 0.434) for profile 3 (**Figure S11C**). Overall, while the model performed well, it had greater difficulty accurately predicting profile 2.

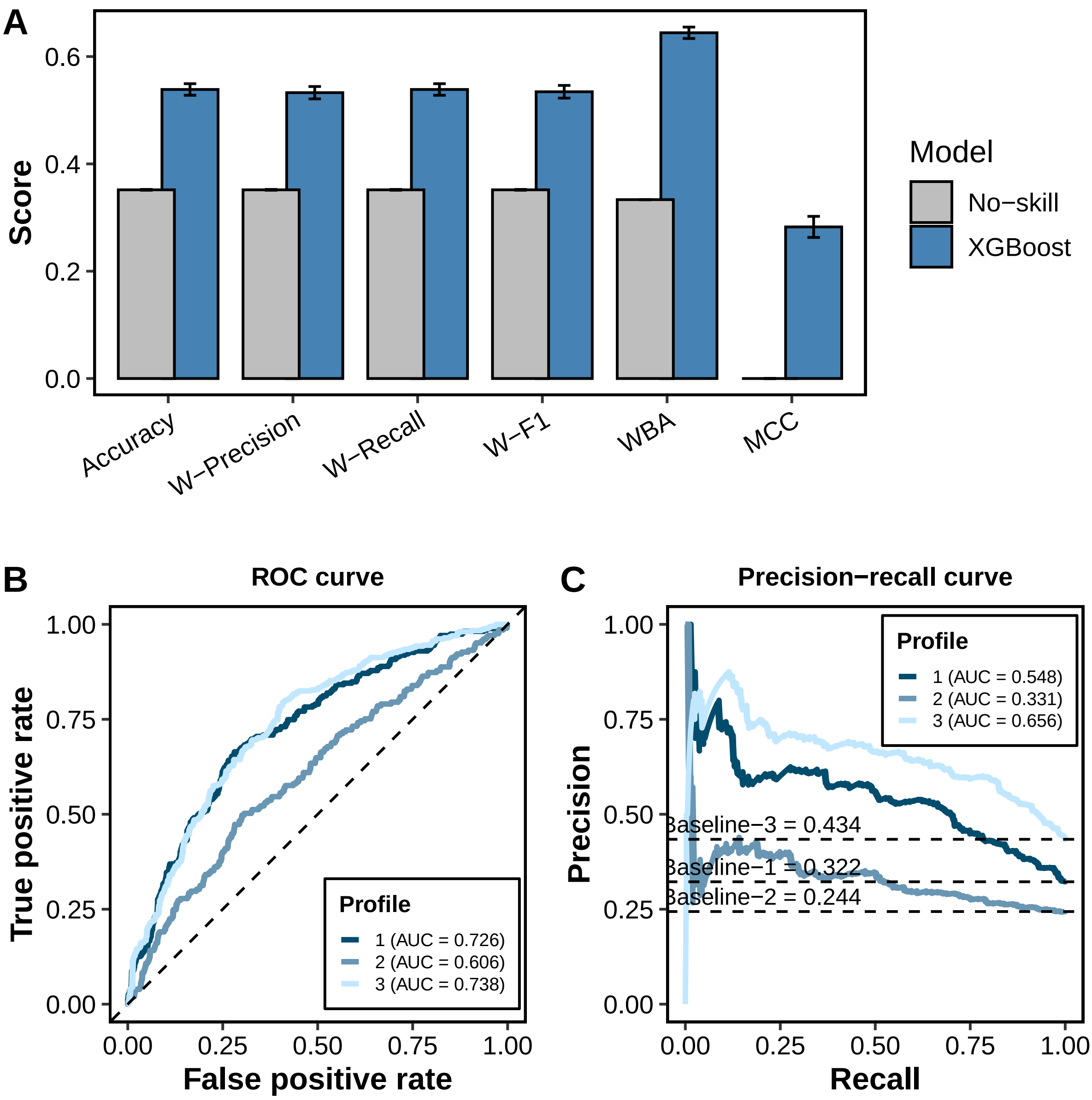

Figure S11: Evaluation of the XGBoost predictive model. **(A)** Comparison between the XGBoost model and a no-skill classifier in different evaluation metrics. The bars and error bars indicate the mean and standard deviation, respectively, of the three-fold models used for nested cross-validation. **(B)** The Area Under the Receiver Operating Characteristic Curve (AUC-ROC) for each profile separately. Colors indicate infection profiles, while the dashed black line indicates random model performance (AUC $= 0.5$). **(C)** Precision-Recall Curves (PR-AUC) for each profile separately. The dashed black lines indicate a no-skill model for each profile, derived from profile prevalence (i.e., a random expectation equal to the fraction of hosts with the infection profile). See **SI note 3** for detailed explanations on model evaluation.

References

1. Sánchez, C. A. *et al.* On the relationship between body condition and parasite infection in wildlife: a review and meta-analysis. *Ecol. Lett.* **21,** 1869–1884 (2018).
2. Durkin, E. S., Luong, L. T. & Bird, J. Mechanisms underlying parasite infection: influence of host body mass and age on chewing louse distribution among brown-headed cowbirds. *Parasitol. Res.* **114,** 4169–4174 (2015).
3. Grear, D. A., Perkins, S. E. & Hudson, P. J. Does elevated testosterone result in increased exposure and transmission of parasites? *Ecol. Lett.* **12,** 528–537 (2009).
4. Klein, S. L. Hormonal and immunological mechanisms mediating sex differences in parasite infection. *Parasite Immunol.* **26,** 247–264 (2004).
5. Cornell, S. J., Bjornstad, O. N., Cattadori, I. M., Boag, B. & Hudson, P. J. Seasonality, cohort-dependence and the development of immunity in a natural host-nematode system. *Proc. Biol. Sci.* **275,** 511–518 (2008).
6. Santin, M., Molokin, A. & Maloney, J. G. A longitudinal study of Blastocystis in dairy calves from birth through 24 months demonstrates dynamic shifts in infection rates and subtype prevalence and diversity by age. *Parasit. Vectors* **16,** 177 (2023).
7. Preston, D. L., Falke, L. P. & Novak, M. Age-prevalence curves in a multi-species parasite community. *Funct. Ecol.* **39,** 91–102 (2025).
8. Mabbott, N. A. The influence of parasite infections on host immunity to co-infection with other pathogens. *Front. Immunol.* **9,** 2579 (2018).
9. Rodgers, M. L. & Bolnick, D. I. Opening a can of worms: a test of the co-infection facilitation hypothesis. *Oecologia* **204,** 317–325 (2024).
10. Ezenwa, V. O. Helminth-microparasite co-infection in wildlife: lessons from ruminants, rodents and rabbits. *Parasite Immunol.* **38,** 527–534 (2016).
11. Rosshart, S. P. *et al.* Wild mouse gut Microbiota promotes host fitness and improves disease resistance. *Cell* **171,** 1015–1028.e13 (2017).
12. Levy, M., Kolodziejczyk, A. A., Thaiss, C. A. & Elinav, E. Dysbiosis and the immune system. *Nat. Rev. Immunol.* **17,** 219–232 (2017).
13. Bernardo-Cravo, A. P., Schmeller, D. S., Chatzinotas, A., Vredenburg, V. T. & Loyau, A. Environmental factors and host microbiomes shape host-pathogen dynamics. *Trends Parasitol.* **36,** 616–633 (2020).

14. Chabé, M., Lokmer, A. & Ségurel, L. Gut Protozoa: Friends or Foes of the Human Gut Microbiota? *Trends Parasitol.* **33,** 925–934 (2017).

15. Gottdenker, N. L., Streicker, D. G., Faust, C. L. & Carroll, C. R. Anthropogenic land use change and infectious diseases: a review of the evidence. *Ecohealth* **11,** 619–632 (2014).

16. Patz, J. A., Graczyk, T. K., Geller, N & Vittor, A. Y. Effects of environmental change on emerging parasitic diseases. *Int. J. Parasitol.* **30,** 1395–1405 (2000).

17. Kiene, F. *et al.* Habitat fragmentation and vegetation structure impact gastrointestinal parasites of small mammalian hosts in Madagascar. *Ecol. Evol.* **11,** 6766–6788 (2021).

18. Patterson, J. E. H. & Ruckstuhl, K. E. Parasite infection and host group size: a meta-analytical review. *Parasitology* **140,** 803–813 (2013).

19. Arneberg, P., Skorping, A., Grenfell, B. & Read, A. F. Host densities as determinants of abundance in parasite communities. *Proc. Biol. Sci.* **265,** 1283–1289 (1998).

20. Begon, M *et al.* A clarification of transmission terms in host-microparasite models: numbers, densities and areas. *Epidemiol. Infect.* **129,** 147–153 (2002).

21. Gajewski, Z. *et al.* Nonrandom foraging and resource distributions affect the relationships between host density, contact rates and parasite transmission. *Ecol. Lett.* **27,** e14385 (2024).

22. Caporaso, J. G. *et al.* Ultra-high-throughput microbial community analysis on the Illumina HiSeq and MiSeq platforms. *ISME J.* **6,** 1621–1624 (2012).

23. Martin, M. Cutadapt removes adapter sequences from high-throughput sequencing reads. *EMBnet.journal* **17,** 10–12 (2011).

24. Callahan, B. J. *et al.* DADA2: High-resolution sample inference from Illumina amplicon data. *Nat. Methods* **13,** 581–583 (2016).

25. Gasser, R. B., Chilton, N. B., Hoste, H & Beveridge, I. Rapid sequencing of rDNA from single worms and eggs of parasitic helminths. *Nucleic Acids Res.* **21,** 2525–2526 (1993).

26. Grandini, M., Bagli, E. & Visani, G. Metrics for multi-class classification: An overview. *arXiv [stat.ML]* (2020).

27. Poisot, T. Guidelines for the prediction of species interactions through binary classification. *Methods Ecol. Evol.* **14,** 1333–1345 (2023).
